# Why Hungarians Have Sex (YSEX?-HSF)

**DOI:** 10.1007/s10508-021-02072-y

**Published:** 2021-11-12

**Authors:** Norbert Meskó, Dóra Szatmári, András Láng, Cindy M. Meston, David M. Buss

**Affiliations:** 1 Institute of Psychology, University of Pécs, Ifjúság útja 6., Pécs, 7624 Hungary; 2 Department of Psychology, University of Texas at Austin, Austin, TX USA

**Keywords:** Sexual motivation, Age and gender differences, Intercourse, Hungarian adaptation

## Abstract

Using the same methodology as Meston and Buss (2007), three studies were conducted on a Hungarian sample (total *N* = 4913) which corroborate previous findings on the universal diversity of sexual motivation. Study 1 (*N* = 2728; 1069 women and 1659 men) identified 197 reasons for having sex based on participants’ free responses. In Study 2 (*N* = 1161; 820 women and 341 men), participants indicated the extent to which each of the 197 reasons had led them to have sexual intercourse. Factor analyses yielded three factors and 24 subfactors. This differed from the original YSEX? four-factor questionnaire. In Study 3 (*N* = 1024; 578 women and 446 men), a reliable and valid 73-item short form version of the YSEX? questionnaire was developed in a Hungarian sample (YSEX?-HSF). In addition to similarities and differences in the factor structure, we found important links between reasons for having sex and age, gender, personality, and mating strategy. For example, number of reasons for having sex tended be higher in younger compared to older participants. Men exceeded women on having sex for novelty-seeking and infidelity opportunities, whereas women exceeded men on having sex for relationship commitment and mate retention. Extraversion and neuroticism were linked with reasons for having sex, and those who pursued a short-term mating strategy reported having sex for a larger variety of reasons.

**Supplementary Information:**

The online version contains supplementary material available at 10.1007/s10508-021-02072-y.

## Introduction

Over the past 40 years, several measures of sexual motivation have been published. Nelson (1978) was the first to develop a self-report measure of sexual motivation. A factor analysis of participants’ responses revealed five distinct reasons for sexual activity: (1) love and affection, (2) pleasure, (3), conformity, (4) recognition-competition, and (5) power (dominance and submission). Nelson separately analyzed male and female participants’ responses and rated 10 sexual variables ranging from the frequency of casual sex to that of reaching orgasm during sexual intercourse. The three most important predictors of sexual variables were conformity, love, and pleasure (in descending order).

Leigh (1989) revealed the following seven reasons for having sex: (1) for pure pleasure, (2) to express emotional closeness, (3) to reproduce, (4) because one’s partner wants to, (5) to please one’s partner, (6) to make a conquest, and (7) to relieve sexual tension. More recently, Hill and Preston (1996) proposed sexual motivation is comprised of the following factors: (1) feeling emotionally valued by one’s partner, (2) expressing feelings of emotional value for one’s partner, (3) obtaining relief from stress, (4) providing one’s partner with nurturance, (5) enhancing one’s feelings of power, (6) experiencing the power of one’s partner, (7) experiencing pleasure, and (8) procreation. Feeling valued by and valuing one’s partner were similar to Nelson’s love and affection factor (*r* < 0.60), while enhancing one’s power and experiencing the partner’s power were identical to Nelson’s dominance and submission factors, respectively. While the questionnaire developed by Hill and Preston (1996) did not include a measure of either conformity or recognition, it did assess stress reduction and procreation as opposed to Nelson’s questionnaire.

Cooper et al. (1998) identified the major dimensions of sexual motivation in a neurological framework based on the dual system of behavior activation (BAS) and behavior inhibition (BIS; see in detail Gray, 1972, 1981). Cooper et al. divided sexual motives into four categories: (1) individual approach motives, e.g., using sex to enhance physical pleasure or positive emotional experience (i.e., enhancement motives); (2) individual avoidance motives, e.g., using sex to cope with threats to self-esteem or to minimize or avoid negative emotions (i.e., coping motives); (3) social approach motives, e.g., using sex to get closer to the partner (i.e., intimacy motives); and (4) social avoidance motives, e.g., using sex to avoid social rejection or to improve social acceptance (i.e., approval motives).

Meston and Buss (2007) pointed out that sexual activity has a much more complex, multifaceted motivational background than previously assumed. In their study, Meston and Buss identified 237 distinct reasons for why men and women engage in sexual intercourse. The reasons were compiled from open ended responses given by individuals aged 17 to 52 years of age, and then administered to over 1500 undergraduate students for the purposes of conducting factor analyses and frequency distributions. Of the 237 distinct reasons reported, 142 loaded onto four primary factors that were equivalent in men and women: Physical Reasons, Goal Attainment Reasons, Emotional Reasons, and Insecurity Reasons. Separate principal component analyses conducted on each of these primary factors revealed between two and four independent subfactors for each of the primary factors.^1^

Recently, Meston et al. (2019) developed a short form of the original YSEX? questionnaire (YSEX?-SF). By reducing the number of items from 144 to 28, Meston et al. developed a version that showed a factor structure identical to that of the original questionnaire, with good internal consistency (Cronbach’s *α* = 0.44 to 0.91), and correlations comparable to the original factors (*r* = 0.84 to 0.94).

Although Meston and Buss (2007) point out the need for research on and development of measures of culture-specific patterns of sexual motives in non-American populations, no such measure has yet been developed to our knowledge. However, there are studies that successfully replicated the factor structure of the original Reasons for Having Sex Questionnaire (YSEX?) in different cultural contexts. In a Norwegian study, 1327 university students rated the 237 translated items of the original pool on which the American questionnaire was based (Kennair et al., 2015). The findings showed that the original 13 factors could be replicated in a more gender-egalitarian country such as Norway. The Turkish version of the YSEX? questionnaire was also based on the translated items of the original pool, which were rated by 401 respondents (234 women; age: *M* = 23.45, range: 18–66; Ozcan et al., 2017). The factor structure of the Turkish version also corresponded to that of the original questionnaire. Gouvernet et al. (2017) translated the original YSEX? questionnaire to French and developed the French version with a sample of 657 respondents (526 women, 131 men; age: *M* = 22.6). Their findings showed that women’s and men’s sexual motivation has different structures. Gouvernet et al. (2017) argued that a reason for this might be the fact that the female network of sexual motives is more compartmentalized and less diffuse than the male network. On the other hand, the male network of sexual motives is more flexible, i.e., motives are more interchangeable for men. Furthermore, Gouvernet et al. (2017) conducted cross-cultural comparisons, since their study also involved an American sample in addition to the French sample. They found more pronounced gender differences in the French sample.

Several researchers highlight the role of social location in multifaceted sexual desire (see Chadwick et al., 2017 for a review) and emphasize that more accurate measurement of a phenomenon requires self-developed questionnaires for that community/subculture rather than translating measures from other societies. Meston and Buss (2007) raised several important questions requiring further research, including whether the factors that they revealed in their North American sample are replicable in different cultures, whether the frequency distribution of motives are culturally specific, whether differences between genders in motives may differ culturally, and whether sexual motives differ according to age and different stages of the life cycle. The primary aim of the present study was to begin examination of these questions in a European sample. A secondary aim of the study was to develop a translated short version of the YSEX? in Hungarian.

We adopted the methodology used by Meston and Buss (2007), and all procedures were kept as closely as possible to that of Meston and Buss in order to allow comparisons to be made between studies. We conducted three studies.

## Study 1: Item Generation

### Method

#### Participants and Procedure

Reasons for having sex were collected from 2728 respondents (age: *M* = 34.84, SD = 9.15, range: 18–65), most of whom (73.81%) completed secondary or tertiary education (13 to 15 years of formal education). The sample included 1069 women (age: *M* = 32.29, SD = 8.79, range: 18–65) and 1659 men (age: *M* = 36.54, SD = 8.99, range: 18–65). Respondents were invited to participate in an anonymous online survey through popular Hungarian websites (http://randiblog.blog.hu, http://elixironline.hu, http://www.marieclaire.hu), social media (e.g., www.facebook.com) and a university mailing list (University of Pécs Faculty of Humanities). The online form was available at www.surveymonkey.com for approximately one month (from 10.10.2012 to 03.11.2012). Respondents were given the following instructions: “Please list all the reasons why you or those you are familiar with or know had sexual intercourse with someone in the past. (You may also add reasons presented in films or books.) We do not expect you to provide complete stories but to list reasons only. Please list as many reasons as you can. Try to describe each reason in a short sentence or in a single word. Enter each reason in a separate field.” Respondents’ gender, age, and the number of completed years in education were also recorded.

### Results

The participants provided a total of 6184 responses. After removing irrelevant responses from the initial pool, the remaining 1231 reasons were subjected to further selection. The first and second authors removed repeated and synonymous responses and compiled a list including 197 reasons. The complete list is not presented here due to its large size.

This list was subsequently presented to another sample (see details in Study 2) in a standard questionnaire format, in which a short description of each reason was accompanied by a 5-point rating scale with scale interval anchors being, None of my sexual experiences (1), A few of my sexual experiences (2), Some of my sexual experiences (3), Many of my sexual experiences (4), and All of my sexual experiences (5). Respondents used the rating scales to indicate how frequently each reason had led them to have sexual intercourse in the past. The questionnaire contained the following instructions: “Several reasons may lead people to have sexual contact (e.g., intercourse) with a partner. The below list enumerates such reasons. Please indicate how frequently each reason led you to have sex with someone in the past. For example, if a reason has never led you to have sex, indicate 1 on the rating scale next to the specific reason. By contrast, if a reason frequently led you to have sex, indicate 5 on the rating scale. If you have never had sexual intercourse, use the rating scales to indicate the likelihood that each reason would lead you to have sex. “I had sex because…”

## Study 2: Psychometric Analysis

### Method

#### Sample and Procedure

Respondents were invited through the above-mentioned online platforms to anonymously complete the 197-item questionnaire. The online form was available for approximately one month (from July 3, 2014 to August 12, 2014). Since respondents were reached through the same channels as those used in Study 1, it is possible that the two samples partly overlapped. A total of 1172 respondents completed the questionnaire. Eleven respondents reported never having had sexual intercourse and were excluded from the sample. The final sample consisted of 1161 respondents (age: *M* = 34.35, SD = 12.39, range: 18–73), which included 820 women (age: *M* = 30.70, SD = 9.88, range: 18–65) and 341 men (age: *M* = 43.14, SD = 13.36, range: 18–73).

The mean age of men was significantly higher than that of women (*F*(1, 1159) = 306.90, *p* < 0.001). The distribution of relationship status was as follows: single: 19.37% (women: 18.80%, men: 19.60%), dating someone (for less than 6 months): 9.64% (women: 10.60%, men: 7.30%), permanent relationship/married 66.13% (women: 66.00%, men: 67.70%), other: 4.86% (women: 4.60%, men: 5.30%. On average, respondents had their first sexual intercourse at the age of 17.58 (SD = 2.66). Men had their first sexual intercourse significantly later (*M* = 18.42, SD = 3.24) than women (*M* = 17.23, SD = 2.30; *F*(1, 1159) = 49.61, *p* < 0.001). The mean number of respondents’ sexual partners in the past was 14.82 (SD = 42.48). Men reported to have had significantly more sexual partners (*M* = 23.84, SD = 51.00) than women (*M* = 11.08, SD = 37.69; *F*(1, 1159) = 22.12, *p* < 0.001).

### Results

#### Item Analysis

The most and least frequent reasons for having sexual intercourse are presented in Supplement 1/A and 1/B.

#### Gender Differences in Reasons for Having Sexual Intercourse

A number of interesting gender differences emerged. Men more than women had sex for sexual variety and had sex when a regular partner was unwilling or unable to do so. Men indicated that they liked the hunt and the risk involved. Women more than men had sex to deepen an existing romantic commitment, to strengthen an emotional bond or attachment, and prevent a partner from leaving. Gender differences in reasons for having sexual intercourse are presented in Supplement 1/C.

#### Principal Component Analysis

Responses to the 197 items were subjected to principal component analyses (PCAs) following the procedure used by Meston and Buss (2007). For data reduction, PCA was deemed for an appropriate method given the data-driven (as opposed to theory driven) nature of our study. Instead of hypothesizing latent constructs that make items correlate (i.e., using EFA), we simply wanted to reduce data, i.e., summarize the many reasons why people have sex into fewer dimensions that can reproduce much of the covariance in the dataset (Abdi & Williams, 2010). Since the items differed in their variances, all PCAs were conducted on *z*-score transformations standardized on the combined sample of men and women. A separate PCA including all 197 items was conducted for each gender group. Direct oblimin rotation with Kaiser normalization was used for the analyses, since related factors were expected. The scree plot showed that the three, four, and five-factor solutions were adequate. KMO measure (0.964) and Bartlett’s Test of Sphericity (approx. *χ*^*2*^(19,306) = 132,526.73; *p* < 0.001) indicated that data were appropriate for factor analysis. The three-factor solution yielded the most consistent pattern of loadings for both men and women, which explained 32% of the total variance in each gender group. The three factors were labeled (1) Personal Goal Attainment, (2) Relational Reasons, (3) and Sex as Coping. As followed by Meston and Buss (2007), the comparability coefficient proposed by Nunnally (1978) was calculated in order to test the correlations between corresponding factors derived from each gender group. The coefficients of correlation between men’s and women’s factors were as follows: Personal Goal Attainment: *r*(46) = 0.27; Relational Reasons: *r*(55) = 0.96; Sex as Coping *r*(43) = 0.31; *p* < 0.001 in all three cases. Since men’s and women’s responses yielded identical factor structures, responses of the overall sample to the 197 items were also subjected to a PCA restricting the number of factors to three and using direct oblimin rotation. The three obtained factors explained 33% of the total variance, and each factor included the expected items the same items as in gender-split analyses (see Table 1).

**Table 1:** Factor analysis of the 197 item YSEX?-H questionnaire in a combined sample of men and women

Item description	Factor		
	Personal goal attainment	Relational reasons	Sex as coping
For the hunt	**.76**	− .06	− .14
I needed a one-night stand	**.73**	.10	− .21
Out of longing for adventure	**.68**	− .22	− .14
It was a challenge	**.63**	− .17	.02
I wanted a new experience	**.61**	− .33	− .08
I took the opportunity	**.61**	− .21	− .10
Out of curiosity	**.60**	− .34	− .09
It was forbidden	**.59**	− .08	.03
I wanted to discover the unknown	**.56**	− .34	− .13
I wanted to boost my ego	**.56**	− .06	.03
I wanted to seek experience	**.56**	− .36	− .16
I love variety	**.55**	− .33	− .09
My partner is not adventurous enough (so I had sex with someone else)	**.55**	.08	− .03
I wanted the excitement of cheating on my partner	**.54**	.04	.04
We hooked up in the heat of the moment	**.51**	− .10	− .05
The person had an exotic appearance	**.51**	− .06	− .07
I wanted to experience a desire for freedom	**.50**	− .26	.04
I wanted to get the other person	**.50**	− .26	.10
I wanted to get some experience	**.50**	− .29	.09
My partner did not want to have sex with me (so I had sex with someone else)	**.49**	.08	− .02
I am addicted to sex	**.48**	− .18	− .00
It was a seduction/I was seduced	**.48**	− .32	− .07
I wanted to show off	**.48**	.10	.09
The other person was acting sexing	**.47**	− .27	.05
Because it was fun	**.46**	− .34	.03
Out of vanity	**.45**	.02	.22
The other person offered himself or herself	**.45**	− .24	.02
For fun	**.44**	.07	.20
I wanted to be cool	**.44**	.06	.17
Out of infidelity	**.43**	.01	.02
My partner was not able to have sex with me (so I had sex with someone else)	**.43**	.07	.00
To justify myself	**.43**	− .10	.24
To get to know my personal boundaries	**.42**	− .29	.10
I wanted to prove myself to others	**.42**	.13	.19
To prove myself	**.41**	− .06	.16
To show my capability	**.40**	− .25	.23
I was under the influence of alcohol	**.40**	− .02	.05
I wanted to boost my self-esteem	**.39**	− .24	.26
It was trendy	**.39**	.13	.14
I wanted to fulfill a need	**.39**	− .29	.08
The person was a good dancer	**.38**	− .17	.03
I wanted to make my partner jealous	**.38**	.08	.28
I wanted to seek revenge	**.37**	.13	.27
I wanted to keep myself in shape	**.37**	− .23	.18
I wanted to demonstrate my power	**.37**	− .00	.26
I wanted to control the other person	**.36**	− .04	.25
Out of friendship	**.36**	− .06	.08
It was a source of inspiration	**.36**	− .25	.06
I wanted to infuriate someone	**.36**	.16	.25
I wanted to increase my prestige	**.35**	.06	.25
It was a fling	**.35**	− .31	.02
I wanted to punish my partner	**.35**	.16	.15
I didn’t want to be the odd one out	**.35**	.12	.20
Out of a desire for possession	**.35**	− .03	.25
I was dissatisfied	**.34**	− .00	.22
I was in an altered state of consciousness	**.34**	− .07	.07
He/she offered me something in exchange for sex	**.34**	.15	.22
I wanted to record it (sound/picture/video)	**.32**	− .05	.01
I wanted to feel appreciated	**.31**	− .14	.29
Instead of talking	**.31**	− .06	.22
It was a bet	.30	.09	.12
It was an initiation	.30	.02	.04
I was under the influence of porn	.29	− .20	.17
I wanted to punish a cheater	.29	.10	.22
I wanted to experience aggression	.29	− .05	.18
I wanted to satisfy my masochistic instincts	.28	− .13	.07
I wanted to humiliate the person	.27	.10	.17
I was having midlife anxiety	.26	.06	.09
I was under the influence of drugs	.22	− .00	.18
I wanted to say „Goodbye”	.22	− .19	.21
I blackmailed him or her/He or she blackmailed me	.22	.10	.20
I wanted to lose my virginity	.21	− .14	.04
Instead of paying or getting paid	.19	.19	.18
Out of shame	.16	.06	.16
It was a religious ritual	.15	.03	.02
Out of mutual appeal	.01	− **.71**	− .10
The person was desirable/sexy	.20	− **.71**	− .15
I wanted to please myself	.10	− **.70**	− .09
I wanted to become one with the other person	− .09	− **.68**	.06
I was in a romantic mood	− .10	− **.66**	.14
I wanted the relationship to grow	− .14	− **.66**	.14
Out of passion	.03	− **.66**	− .04
I wanted pleasure	.23	− **.65**	− .10
I wanted to intensify the commitment	− .20	− **.65**	.35
I wanted to deepen the relationship	− .18	− **.64**	.23
Because of sexual desire	.23	− **.63**	− .16
I wanted to be attached to the person	− .23	− **.62**	.30
I was horny	.19	− **.62**	− .02
I wanted to get on the same wavelength as the other person	− .07	− **.62**	.24
It was a pleasant pastime	.22	− **.62**	− .05
I wanted to satisfy my partner	.07	− **.61**	.05
It was a way to express my feelings	− .12	− **.60**	.20
The person had an attractive personality	.23	− **.60**	− .06
I wanted to get closer to the person	.00	− **.60**	.21
I wanted to be satisfied	.24	− **.60**	− .07
I was in love	− .27	− **.60**	.06
I wanted to have an orgasm	.23	− **.59**	− .07
I was seeking emotional closeness (i.e., intimacy)	− .08	− **.57**	.24
I wanted to recharge	.18	− **.57**	.05
I love sex	.34	− **.57**	− .25
To seek pleasure	.21	− **.57**	− .14
The person was too ‘‘hot’’ (sexy) to resist	.23	− **.55**	− .10
Out of affection	− .14	− **.55**	.09
I needed it	.30	− **.52**	.02
I wanted to care for the other person	− .10	− **.51**	.39
The person’s touch was pleasant	.08	− **.49**	− .07
The person smelled good	.17	− **.49**	.02
We were made for each other	− .17	− **.48**	.077
I wanted to conquer the other person	.31	− **.48**	.08
I wanted to feel restored	.23	− **.47**	.10
The person had an attractive body	.44	− **.45**	− .15
I liked the person	.19	− **.45**	.13
I wanted to relax	.16	− **.45**	.20
I wanted to be/feel happy	.08	− **.45**	.01
Out of trust	− .17	− **.45**	.17
I wanted to act out a fantasy	.40	− **.44**	− .07
I wanted to cheer up the other person	.03	− **.44**	.43
I wanted to make my partner happy	− .07	− **.43**	.06
It was an inner drive (I couldn’t control myself)	.32	− **.42**	.05
I wanted to celebrate	.07	− **.42**	.28
It seemed like the next step in the relationship	.00	− **.42**	.10
I wanted to try out new sexual techniques or positions	.33	− **.42**	.03
I wanted to know how it feels to get acquainted with him or her	.24	− **.41**	.13
It was a special occasion/situation	.39	− **.41**	− .02
The person had an attractive voice	.29	− **.40**	.01
I wanted to have sex in an unusual place or situation	.38	− **.39**	.01
Because of commitment	− .17	− **.38**	.37
I wanted to spiritually merge with the other person	.01	− **.35**	.10
I wanted to express myself	.25	− **.35**	.18
I wanted to reduce stress	.22	− **.35**	.23
I wanted to increase my self-knowledge	.32	− **.32**	.19
I wanted the physical exercise	.25	− .29	.17
I gave him or her intercourse as a gift	.09	− .20	.16
I didn’t want to lose the person	− .09	− .18	**.67**
I wanted to avoid a fight/conflict	− .00	.01	**.67**
I wanted to save the relationship	− .06	− .12	**.65**
Out of duty	− .10	.01	**.62**
I was afraid that my partner would leave me if I didn’t have sex with him/her	− .09	− .10	**.60**
Out of remorse	.05	.01	**.58**
I wanted to manage a conflict	.04	− .21	**.58**
I wanted to retain the relationship	− .07	− .23	**.57**
I wanted to meet the person’s expectations	.09	− .09	**.57**
I wanted to express my forgiveness	− .02	− .20	**.55**
I wanted to comfort the other person	.09	− .14	**.54**
I wanted to appease the other person	− .01	− .26	**.54**
I wanted to improve the relationship	− .07	− .35	**.53**
I wanted to give in	.00	− .04	**.52**
Out of habit/routine	− .03	− .17	**.50**
Out of fear	− .01	.07	**.48**
Out of compassion	.13	.07	**.48**
I wanted to soothe myself/my partner	.10	− .30	**.46**
I wanted comfort	.25	− .13	**.46**
I wanted support	.03	− .32	**.46**
I wanted to avert my partner’s suspicion	.21	.05	**.45**
Out of despair	.25	.06	**.44**
I wanted to seek safety	.00	− .15	**.43**
I wanted to decrease sadness	.18	− .24	**.43**
I wanted to apologize	.02	− .13	**.43**
I wanted to make peace with the other person	.01	− .15	**.42**
I wanted to reduce anxiety	.15	− .08	**.41**
It was marital duty	− .07	.00	**.39**
I wanted to belong to someone	.03	− .35	**.39**
Out of sympathy	.08	− .06	**.38**
I wanted to distract attention	.19	− .10	**.37**
Out of gratitude	.10	− .18	**.37**
I wanted to influence the other person	.21	.00	**.36**
I wanted to submit myself	.08	− .01	**.36**
I wanted to forget about my problems	.23	− .17	**.36**
It was a favor	.19	.01	**.36**
I wanted to improve my mood	.26	− .30	**.36**
Because I lacked love	.26	− .15	**.34**
I wanted to benefit from it	.24	.15	**.34**
I wanted to decrease my anger	.25	− .11	**.33**
Due to lack of privacy	.25	− .11	**.33**
I wanted to decrease loneliness	.31	− .18	**.33**
It was a way to reach my goal	.30	.10	**.33**
I wanted to control the person	.31	− .14	**.33**
Out of rivalry (to show somebody I’m better than him/her)	.27	.01	**.32**
Out of boredom	.29	− .14	**.32**
I wanted to profit from it	.20	.24	**.32**
I wanted to prove something to my partner	.21	− .16	**.32**
Out of envy	.26	.14	**.31**
For livelihood	.05	.15	.29
For money	.18	.21	.29
I wanted to gain financial interest	.23	.25	.29
I wanted to escape from the previous relationship			
I couldn’t sleep			
I wanted to relieve pain			
I felt estranged from a partner			
I couldn’t say no			
It was a rebellion against parental rules			
I was influenced by a movie			
I hoped it would lead to advancement			
Due to a threat			
I wanted to have a child			
Due to violence			
For career advancement			
Initial eigenvalues	44.34	11.20	6.52
Variance explained (%)	22.51	5.68	3.31

Strongest factor loadings above |.30 | are bolded

#### Subfactor Analysis and Scale Construction

Because each of the three factors contained a large (i.e., 60, 56, 49 items for Personal Goal Attainment, Relational Reasons, and Sex as Coping, respectively) and quite heterogeneous number of different reasons, separate PCAs were conducted on items within each of the three broad domains. KMO measures (0.960, 0.973, and 0.957 for Personal Goal Attainment, Relational Reasons, and Sex as Coping, respectively) and Bartlett’s Tests of Sphericity (approx. *χ*^*2*^(1770) = 32,286.66, approx. *χ*^*2*^(1540) = 35,283.93, and approx. *χ*^*2*^(1176) = 24,965.48 for Personal Goal Attainment, Relational Reasons, and Sex as Coping, respectively; all *p*s < 0.001) Using initial eigenvalue = 1 as threshold, PCAs revealed a different number of subfactors within each factor. For Personal Goal Attainment 11 subfactors (accounting for 57.70% of the total variance) emerged, out of which three factors were omitted because of inconsistent item meanings. Factor 6—explaining 2.48% of the total variance—contained items “Instead of talking,” “The other person offered himself or herself,” “Out of friendship,” and “I wanted to keep myself in shape.” Factor 9—explaining 1.91% of the total variance—contained items “It was a source of inspiration,” “The person was a good dancer,” and “To get to know my personal boundaries” with loadings above |.30|. Factor 10—explaining 1.79% of total variance—contained items “I wanted to record it (sound/picture/video)” and “I was dissatisfied” with loadings above |.30|. Thus, the final number of subfactors for Personal Goal Attainment was eight (Table 2).

**Table 2:** Subfactor analysis of personal goal attainment for engaging in sexual intercourse

Item description	Factor							
	Seeking novelty	Conformity	Infidelity	Impulsiveness	Revenge	Seeking sensation	Control and power	Boosting self-esteem
**I wanted new experience**	**.50**	.07	.09	− .02	.03	− .27	− .05	− .16
**I love variety**	**.49**	− .02	.11	.01	− .01	− .17	.10	− .05
**It was forbidden**	**.44**	.00	.29	.10	.22	− .12	.01	.03
**I wanted to get some experience**	**.43**	.17	− .02	− .02	.08	− .11	− .05	− .25
**I wanted to experience a desire for freedom**	**.43**	.04	.09	.11	.07	− .08	− .01	− .12
**Out of curiosity**	**.40**	.03	.02	.02	− .01	− .38	− .00	− .15
**For the hunt**	**.39**	.12	.15	.19	.03	− .25	.19	.02
**Because I was addicted to sex**	**.39**	− .03	.13	.06	.02	.02	.21	.03
**It was a challenge**	**.36**	.14	.11	.06	.04	− .17	.17	− .12
**The other person was acting sexing**	**.36**	.04	.08	.17	.00	− .10	.22	.11
**Because it was fun**	**.36**	− .02	− .02	.29	.01	− .03	.09	− .06
**I wanted to get the other person**	**.35**	.01	.04	.04	.09	− .20	.24	− .14
**It was trendy**	− .03	**.76**	.00	− .01	.04	− .04	− .08	.02
**I didn’t want to be the odd one out**	− .04	**.74**	.03	.05	.07	.05	− .20	− .17
**I wanted to be cool**	.19	**.73**	− .01	.11	− .10	.09	.16	− .05
**I wanted to show off**	.09	**.59**	− .00	.04	.18	.03	− .05	− .08
**I wanted to prove myself to others**	− .09	**.50**	.08	.07	− .00	− .06	.13	− .18
For fun	.02	.28	.10	.19	.24	.14	.15	.09
**My partner did not want to have sex with me (so I had sex with someone else)**	− .09	− .01	**.95**	.01	− .04	.09	− .03	− .00
**My partner was not able to have sex with me (so I had sex with someone else)**	− .10	− .06	**.88**	− .01	− .05	.12	− .03	− .02
**My partner is not adventurous enough (so I had sex with someone else)**	.04	.08	**.85**	− .07	− .02	.03	− .02	.02
**Out of infidelity**	.11	− .10	**.58**	.03	.15	− .17	.06	− .02
**For the excitement of cheating on my partner**	.30	.05	**.54**	− .00	.14	− .09	.04	.03
**I was under the influence of alcohol**	− .08	.00	− .01	**.86**	.05	.04	− .04	− .01
**I was in an altered state of consciousness**	.03	.04	− .04	**.81**	− .05	.11	.07	− .06
**We hooked up in the heat of the moment**	− .05	− .04	− .02	**.69**	.07	− .25	− .08	.04
**I needed a one-night stand**	− .02	.15	.15	**.42**	.14	− .36	− .09	.10
**I wanted to infuriate someone**	.00	.01	− .08	.01	**.86**	.09	− .03	.03
**I wanted to take revenge**	.05	− .04	− .02	.04	**.79**	− .01	.07	− .01
**I wanted to make my partner jealous**	.07	.02	.06	.06	**.69**	.06	− .03	− .06
**I wanted to punish my partner**	− .19	− .01	.04	− .06	**.65**	− .01	.02	.04
*Instead of talking*	− .10	.01	.07	.07	.15	.01	.01	− .10
*The other person offered himself or herself*	.31	.11	.03	.13	.00	− .09	.13	.13
*Out of friendship*	− .01	.16	.00	− .03	.13	− .22	− .05	− .05
*I wanted to keep myself in shape*	.22	.03	.05	.03	.01	.11	.25	− .09
**I wanted to seek experience**	.07	− .04	.04	.04	− .06	− **.63**	.01	− .16
**It was a seduction/I was seduced**	.01	− .06	− .03	.06	.06	− **.62**	.11	− .07
**I took the opportunity**	.16	.09	− .03	.13	.07	− **.55**	.00	.04
**Out of longing for adventure**	.20	.03	.09	.10	.00	− **.55**	− .02	− .09
**To discover the unknown**	− .00	− .06	− .01	.16	.03	− **.52**	.06	− .02
**The person had an exotic appearance**	.28	.00	.08	− .07	.08	− **.48**	− .14	− .13
**I wanted to seek experience**	− .16	.20	.12	.03	− .08	− **.42**	.05	.03
**I wanted to control the other person**	.07	− .13	− .02	.05	.06	.00	**.74**	− .08
**I wanted to demonstrate my power**	− .01	− .03	.04	.02	.07	.07	**.71**	− .14
**Out of a desire for possession**	− .11	− .08	.03	.14	.15	− .11	**.45**	− .22
**I wanted to increase my prestige**	− .02	.35	.02	− .05	− .03	− .00	**.41**	.01
He/she offered me something in exchange for sex	− .04	.33	.03	− .17	.18	− .22	.38	.35
Out of vanity	− .03	.20	.01	.04	.22	− .15	.26	− .23
*It was a source of inspiration*	− .01	− .07	.10	.00	.11	− .22	.04	− .05
*The person was a good dancer*	.06	.09	.07	.28	− .01	− .11	.05	.18
*To get to know my personal boundaries*	.15	− .08	.04	.05	.14	− .17	.00	− .33
*I wanted to record it (sound/picture/video)*	.06	− .04	.05	.01	.10	− .03	.09	− .04
*I was dissatisfied*	− .16	.20	.19	.05	.10	− .22	.11	− .08
*I wanted to fulfill a need*	.19	− .03	.18	.05	.07	− .17	.02	− .17
**To prove myself**	− .09	.14	.08	.08	− .01	− .11	.08	**− .63**
**I wanted to boost my self-esteem**	.22	.11	.03	.01	.07	− .01	.08	**− .60**
**I wanted to feel appreciated**	− .15	.14	.03	− .00	.01	− .17	.19	**− .52**
**To justify myself**	.23	.13	.04	.03	.11	.06	.17	**− .52**
**To boost my ego**	− .05	.24	.02	.12	.11	− .16	.12	**− .50**
**To show my capability**	.30	.05	.04	− .02	.01	− .00	.24	**− .41**
Initial eigenvalues	17.80	2.90	2.63	2.13	1.82	1.41	1.25	1.01
Variance explained (%)	29.66	4.78	4.39	3.55	3.03	2.34	2.08	1.69

Loadings of items to their final factors are bolded. Items bolded were included in the composite scoring. Items of the omitted factors are italicized

For Relational Reasons, 9 subfactors (accounting for 55.47% of the total variance) emerged (Table 3). For Sex as Coping, 8 subfactors (accounting for 54.48% of the total variance) emerged, out of which one factor was omitted because of inconsistent item meanings. Factor 7—explaining 2.56% of total variance—contained items “Out of fear,” “Out of remorse,” and “I wanted to distract attention” with loadings above |.30|. This left the Sex as Coping factor with seven subfactors (Table 4). One item (“It was marital duty”) was omitted because it was deemed as redundant to a more broadly worded item (“Out of duty”) with considerably similar factor loading.

**Table 3:** Subfactor analyses of relational reasons for engaging in sexual intercourse

Item description	Factor								
	Sexual desire	Commitment	Care	Physical attraction	Relaxation	Intimacy	Excitement	Self-affirmation	Happiness seeking
**I wanted to have an orgasm**	**.81**	.01	.09	− .08	.08	− .04	− .03	.01	.01
**Because of sexual desire**	**.77**	− .05	.02	.05	.04	.15	− .00	.02	− .03
**I wanted pleasure**	**.75**	− .05	.01	.06	.03	.05	− .02	.13	.01
**I love sex**	**.72**	− .13	− .03	.08	.09	.14	− .06	.01	− .04
**I wanted to be satisfied**	**.71**	.18	.01	− .08	.10	− .12	− .15	− .10	.07
**I wanted to please myself**	**.64**	.05	.05	.02	.04	.14	.07	.09	.14
**To seek pleasure**	**.56**	.07	− .19	.04	.16	− .04	.07	− .04	.30
**I was horny**	**.52**	.19	.00	.07	.07	.01	− .29	− .14	.05
**I needed it**	**.52**	− .02	.07	.05	.16	− .04	− .16	.14	− .10
**It was a pleasant pastime**	**.35**	.17	.10	.25	.27	− .00	− .04	− .09	− .01
**I wanted to satisfy my partner**	**.34**	.21	.14	− .01	− .08	.08	− .27	− .07	.29
**It seemed like the next step in the relationship**	− .05	**.69**	− .08	.16	.18	− .00	.05	− .09	− .11
**I wanted to deepen the relationship**	.04	**.64**	.09	− .00	.08	.18	− .05	.07	− .03
**I wanted the relationship to grow**	.05	**.64**	.01	.03	.08	.24	− .06	.09	− .07
**Because of commitment**	− .02	**.54**	.13	− .23	.03	− .08	− .10	.09	.26
**I wanted to get closer to the person**	.10	**.42**	.16	.20	− .08	− .10	− .08	.25	.09
**I wanted to be attached to the person**	.16	**.40**	.26	− .01	− .02	.15	.03	.13	.11
**I wanted to intensify the commitment**	.09	**.39**	.25	− .04	.03	.10	.04	.37	.08
**It was a way to express my feelings**	.09	**.39**	− .08	.07	.17	.15	.06	.09	.20
**Out of mutual appeal**	.31	**.34**	− .12	.23	− .05	.29	− .03	.00	.06
**I wanted to care for the other person**	.02	.12	**.51**	.09	.13	.05	− .01	.11	.13
**I wanted to cheer up the other person**	− .05	.02	**.45**	.12	.24	.03	− .18	.15	.08
**I wanted to celebrate**	− .05	.07	**.38**	.05	.29	.14	− .33	− .11	.05
**The person had an attractive body**	.12	− .04	− .05	**.66**	.09	− .04	− .13	− .09	.08
**The person smelled good**	− .15	.08	.04	**.63**	.21	.05	− .07	− .07	.11
**The person had an attractive personality**	.12	.15	.03	**.61**	− .10	.06	− .03	.15	.02
**The person had an attractive voice**	− .05	− .10	.16	**.59**	.11	.05	− .10	.08	− .03
**The person was too ‘‘hot’’ (sexy) to resist**	.02	.15	− .29	**.41**	.05	.09	− .24	.06	.26
**I liked the person**	.18	.17	.35	**.39**	− .08	− .24	.01	.10	.07
**The person was a desirable/sexy**	.34	.25	− .14	**.37**	− .01	.14	− .12	− .04	.09
I wanted to know how it feels to get acquainted with him or her	.03	.27	.09	.34	.06	− .17	.04	.29	− .04
**I wanted to feel restored**	.06	.03	− .03	.01	**.79**	− .03	− .03	.01	.05
**I wanted to reduce stress**	.06	.09	− .07	− .06	**.72**	− .10	− .03	.09	− .00
**I wanted to recharge**	.14	.06	− .03	.07	**.68**	.03	.03	.03	.08
**I wanted to relax**	.07	− .05	.25	.09	**.61**	.08	− .02	.07	− .15
**I was in love**	.16	.10	.11	− .00	− .06	**.71**	− .03	− .02	.08
**We were made for each other**	− .10	.18	− .20	− .07	.05	**.46**	− .16	.14	.33
**Out of passion**	.37	.06	.00	.09	.02	**.45**	− .06	.16	− .05
**Out of affection**	.22	− .00	.40	.11	− .01	**.41**	.18	.06	.06
**I was in a romantic mood**	.03	.16	.13	.17	.11	**.38**	− .05	.20	.05
**I wanted to have sex in an unusual place or situation**	.12	− .10	.04	.07	.13	.14	**− .55**	.16	− .08
**It was a special occasion/situation**	.01	.05	.02	.25	.01	.01	**− .54**	.13	− .00
**I wanted to try out new sexual techniques or positions**	.16	− .14	.29	.11	.14	.04	**− .49**	− .02	.05
**I wanted to act out a fantasy**	.18	.06	− .16	.03	.22	− .07	**− .48**	.09	.12
**It was an inner drive (I couldn’t control myself)**	.26	.07	− .05	.15	.06	− .09	**− .35**	.11	.00
**I wanted to conquer the other person**	.18	.26	.03	.27	− .17	− .15	**− .32**	.25	.02
**I wanted to spiritually merge with the other person**	− .10	− .04	− .14	− .05	.09	.22	− .09	**.69**	− .02
**I wanted to express myself**	.01	− .02	.10	.06	.13	− .14	− .19	**.57**	.00
**To get on the same wavelength with the other person**	.11	.25	.19	.06	.06	.01	.12	**.50**	.09
**I wanted to increase my self-knowledge**	.08	− .03	.18	.01	.15	− .25	− .21	**.45**	.08
**I wanted to become one with the other person**	.21	.11	.02	.04	.30	.36	.07	**.44**	.04
**I was seeking emotional closeness (i.e., intimacy)**	.14	.20	.14	.12	.17	− .00	.25	**.38**	.10
**I wanted to make my partner happy**	− .03	− .05	.12	− .01	− .04	.11	− .13	− .02	**.74**
**I wanted to be/feel happy**	.26	− .07	− .08	.07	.10	− .13	.17	.15	**.59**
**The touch of the person was pleasant**	.05	− .14	− .09	.41	.03	.07	.08	.04	**.56**
**Out of trust**	− .12	.20	.27	− .00	.16	.06	.00	− .10	**.46**
Initial eigenvalues	20.07	2.78	2.53	1.71	1.42	1.35	1.19	1.14	1.05
Variance explained (%)	35.85	4.97	4.52	3.05	2.53	2.41	2.13	2.03	1.88

Loadings of items to their final factors are bolded. Items bolded were included in the composite scoring

**Table 4:** Subfactor analyses of sex as coping for engaging in sexual intercourse

Item description	Factor						
	Mitigating emotional deficit	Compulsion and avoidance	Utilitarianism	Coping with relational conflicts	Submissiveness	Coping with partner’s emotional demands	Mate retention
**I wanted to decrease loneliness**	**.71**	.03	.03	.11	.08	.03	− .12
**Because I lacked love**	**.69**	.11	− .02	.09	.05	.03	− .06
**I wanted to decrease sadness**	**.68**	− .01	− .09	− .09	− .11	.14	− .05
**I wanted to improve my mood**	**.68**	.00	.04	− .10	− .01	.00	− .09
**I wanted comfort**	**.65**	.08	.04	.03	− .10	.07	− .12
**I wanted to forget about my problems**	**.62**	− .01	.07	− .26	.15	− .05	.14
**I wanted support**	**.60**	.03	.01	.11	.08	.14	− .25
**Out of despair**	**.57**	.01	.14	− .07	.22	− .07	.12
**I wanted to belong someone**	**.54**	.02	.02	.19	.13	.10	− .35
**I wanted to reduce anxiety**	**.53**	− .02	− .10	.01	− .05	.14	− .00
**Out of boredom**	**.40**	.06	.12	− .21	− .23	.19	.07
**I wanted to soothe myself/my partner**	**.37**	− .03	− .06	− .20	− .03	.24	− .18
Due to lack of privacy	.25	.17	.20	− .18	− .15	.22	.09
It was marital duty	− .11	**.**83	− .05	− .00	.01	− .06	− .00
**Out of duty**	− .09	**.75**	.02	.03	.08	.06	− .10
**Out of habit/routine**	.15	**.68**	− .03	− .03	.07	.05	− .04
**I wanted to avert my partner’s suspicion**	.09	**.38**	.15	− .13	− .23	− .03	− .02
**I wanted to avoid a fight/conflict**	− .01	**.38**	.140	− .19	− .14	.10	− .20
I wanted to meet the person’s expectations	.21	.30	.04	.05	.15	.12	− .27
**I wanted to profit from it**	− .11	.02	**.72**	.12	− .04	.05	.02
**It was a way to reach my goal**	− .02	.03	**.69**	− .19	.12	.07	.02
**I wanted to benefit from it**	− .04	− .08	**.63**	.03	.18	.13	− .02
**I wanted to influence the other person**	.10	.065	**.57**	− .22	.13	− .04	− .01
**Out of envy**	.015	.002	**.55**	.09	− .19	.03	− .05
**Out of rivalry (to show somebody I’m better than him/her)**	.18	.14	**.37**	.08	− .28	− .04	− .20
**I wanted to control the person**	.32	.06	**.32**	− .14	− .10	.04	− .07
**I wanted to apologize**	− .12	.06	.04	**− .71**	.18	.04	− .07
**I wanted to make peace with the other person**	− .09	.12	− .02	**− .70**	.07	− .00	− .11
**I wanted to appease the other person**	.00	.03	− .05	**− .54**	− .12	.23	− .32
**I wanted to manage a conflict**	.11	.07	− .01	**− .48**	− .16	.10	− .27
**I wanted to decrease my anger**	.34	− .07	.23	**− .44**	− .04	− .08	.07
**I wanted to express my forgiveness this way**	.08	.03	.06	**− .43**	− .12	.19	− .22
**I wanted to submit myself**	.07	.14	.10	− .07	**.53**	.07	− .02
**I wanted to seek safety**	.35	− .01	.02	− .06	**.42**	.04	− .17
**I wanted to prove sg to my partner**	.11	.09	.07	− .33	**.34**	.05	− .13
**I wanted to give in**	− .00	.21	.16	.03	**.33**	.27	− .06
**It was a favor**	.00	.06	.15	.10	− .07	**.73**	.01
**Out of sympathy**	.05	.04	− .10	− .130	.27	**.67**	.13
**Out of gratitude**	− .06	− .21	.14	− .11	.01	**.59**	− .22
**Out of compassion**	.09	.24	.07	.10	− .04	**.55**	.10
**I wanted to comfort the other person**	.25	.09	− .05	− .17	− .21	**.42**	− .12
*Out of fear*	.03	− .02	.05	.055	.18	.02	− .14
*Out of remorse*	.04	.23	.04	− .20	− .12	.11	− .11
*I wanted to distract attention*	.31	.03	.07	− .26	− .01	.07	.12
**I wanted to retain the relationship**	.00	.10	.03	− .09	.02	− .02	**− .75**
**I wanted to save the relationship**	− .04	.15	.04	− .13	− .05	− .02	**− .70**
**Not to lose the other person**	.13	.10	− .01	− .02	.07	.04	**− .69**
**I wanted to improve the relationship**	.07	.07	− .02	− .18	− .05	.041	**− .64**
**I was afraid that my partner would leave me (if I didn’t have sex with him/her)**	.14	.04	.13	− .03	.34	− .11	**− .49**
Initial eigenvalues	15.00	2.58	2.20	1.72	1.61	1.30	1.04
Variance explained (%)	30.61	5.26	4.49	3.50	3.28	2.66	2.12

Strongest factor loadings above | .30 | are bolded. Items bolded were included in the composite scoring. Items of the omitted factor are italicized

Subfactors of the *Personal Goal Attainment* factor were labeled as follows: Novelty Seeking, Conformity, Infidelity, Impulsiveness, Revenge, Sensation Seeking, Control and Power, Self-Esteem Boost. Subfactors of the *Relational Reasons* factor were labeled as follows: Sexual Desire, Commitment, Care, Physical Attraction, Relaxation, Intimacy, Excitement, Self-Affirmation, Happiness Seeking. Subfactors of the *Sex as Coping* factor were labeled as follows: Emotional Need Satisfaction, Compulsion and Avoidance, Utilitarianism, Coping with Relational Conflicts, Submission, Dealing with Partner’s Emotional Needs, Mate Retention. Within the respective factors, each subfactor was relatively homogeneous in content while considerably distinct from the other subfactors. This suggests that each subfactor taps not only a statistically but a psychologically and functionally distinct category.

Items with a factor loading not greater than 0.30 were removed from each subfactor. For this reason, 14 items were removed from the *Personal Goal Attainment* factor and 4 items from the *Sex as Coping* factor. One item was removed from the *Relational Reasons* factor because the content of the item “For the emotions I feel when discovering a new partner” was deemed inconsistent with the other items of the Physical Attraction subfactor. (see Tables 2, 3, 4).

Internal consistency of the resulting 144-item Hungarian version of the YSEX? (YSEX?-H) was tested by calculating Cronbach’s *α* coefficients of the subfactors and composite factors for each gender group and for the overall sample (see Supplement 2).

#### Composite Analysis

*Gender differences in sexual motivation* In order to assess gender differences in composite scores, the samples of women and men were compared on each factor and subfactor by independent samples *t*-tests. Table 5 shows that significant differences were found between the two samples on some of the factors as well as on some of the subfactors (at three significance levels). A gender difference was found on the *Personal Goal Attainment* factor, on which men scored significantly higher than women (*p* < 0.001). No significant difference was found on the *Relational Reasons* factor (although differences were found on some of the subfactors, which are addressed below). The *Sex as Coping* factor also revealed a significant gender difference, on which women scored higher than men (*p* < 0.005).

**Table 5:** Gender differences in 144 items YSEX?-H factor and subfactor scores

Factor	Subfactor	Women		Men		*t*	*p*	*d*
		Mean	SD	Mean	SD			
**Personal goal attainment**		**75.30**	**23.19**	**83.38**	**27.17**	**5.14**	** < .001**	**− .31**
	**Novelty seeking**	**22.33**	**9.18**	**25.64**	**1.46**	**5.36**	** < .001**	**− .34**
	**Conformity**	**5.72**	**1.76**	**6.48**	**2.63**	**5.73**	** < .001**	**− .34**
	**infidelity**	**6.39**	**2.66**	**7.65**	**3.71**	**6.51**	** < .001**	**− .39**
	**Impulsiveness**	**7.02**	**3.09**	**7.53**	**3.19**	**2.57**	**.010**	**− .16**
	Revenge	4.75	1.64	4.67	1.65	− .73	.463	.05
	**Sensation seeking**	**13.06**	**5.02**	**14.74**	**5.45**	**5.06**	** < .001**	− **.32**
	**Control and power**	**5.28**	**2.04**	**5.64**	**2.47**	**2.54**	**.011**	− **.16**
	Self-esteem boost	1.74	4.89	11.03	5.03	.889	.374	− .06
Relational reasons		16.79	42.25	156.88	41.67	− 1.44	.150	.09
	Sexual desire	38.37	11.06	39.15	1.57	1.10	.273	− .07
	**Commitment**	**28.01**	**8.75**	**25.13**	**8.40**	− **5.16**	** < .001**	**.34**
	**Physical attraction**	**18.42**	**6.54**	**19.59**	**6.47**	**2.79**	**.005**	− **.18**
	Relaxation	9.03	3.98	8.62	3.95	− 1.62	.104	.10
	**Intimacy**	**17.90**	**4.57**	**16.82**	**4.82**	− **3.62**	** < .001**	**.23**
	Excitement	14.40	5.30	14.72	5.53	.932	.352	− .06
	**Self-affirmation**	**13.94**	**5.23**	**12.99**	**5.17**	− **2.85**	**.004**	**.18**
	Care	6.47	2.77	6.12	2.84	− 1.95	.052	.12
	**Happiness seeking**	**14.25**	**3.84**	**13.75**	**3.79**	− **2.00**	**.045**	**.13**
**Sex as coping**		**68.95**	**21.21**	**65.64**	**21.49**	− **2.41**	**.016**	**.15**
	**Mitigating emotional deficit**	**21.05**	**8.50**	**19.46**	**7.82**	− **2.96**	**.003**	**.19**
	Compulsion and avoidance	6.37	2.77	6.10	2.59	− 1.58	.114	.10
	Utilitarianism	8.57	2.54	8.48	2.66	− .57	.571	.04
	Coping with relational conflicts	1.22	4.11	9.99	3.94	− .9	.368	.06
	**Submissiveness**	**6.85**	**2.84**	**6.35**	**2.63**	− **2.79**	**.005**	**.18**
	**Coping with partner’s emotional demands**	**6.43**	**2.11**	**6.75**	**2.84**	**2.11**	**.035**	− **.13**
	**Mate retention**	**9.45**	**4.40**	**8.52**	**3.75**	− **3.43**	**.001**	**.23**

*df* = 1159 for each *t*-test. Significant differences are bolded

An analysis of men’s and women’s scores on the subfactors further revealed gender differences in sexual motivation. Men attained a higher mean score on the *Personal Goal Attainment* factor because they scored higher on all of its subfactors where a significant difference was found, namely, on Novelty Seeking, Conformity, Infidelity, Impulsiveness, Sensation Seeking, and Control and Power. The *Relational Reasons* factor revealed no significant gender difference, probably because the significant differences found on five of its subfactors counterbalanced each other. The only subfactor on which men scored significantly higher than women was Physical Attraction (*p* < 0.05). By contrast, women scored higher on the Commitment, Intimacy, Self-Affirmation and Happiness Seeking subfactors (*p* < 0.001, *p* < 0.001, 0.005, and 0.05, respectively). Women’s higher mean score on the *Sex as Coping* factor was due to their higher scores on the Submission and Mate Retention subfactors (*p* < 0.05 in both cases), whereas men scored higher on the Dealing with Partner’s Emotional Needs subfactor (*p* < 0.005). No significant difference was found on the other four subfactors. Correlations between age and the primary factors and subfactors of YSEX-H are presented in Supplement 3.

## Study 3: Short Version, Personality, Sociosexuality

### Method

#### Development of a Short Version

*Sample and procedure* Respondents were invited through the above-mentioned online platforms to anonymously complete a questionnaire. The online form was available for about a month (from October 10, 2014 to November 8, 2014). Since respondents were reached through the same channels as those used in Studies 1 and 2, it is possible that the samples partly overlapped. A total of 1032 respondents completed the questionnaire. Since only 8 respondents reported never having engaged in sexual intercourse, they were excluded from the sample. Responses of the remaining 1024 respondents were analyzed in Study 3 (age: *M* = 33.81, SD = 10.81, range: 18–80). The sample included 578 women (age: *M* = 30.47, SD = 8.75, range: 18–74) and 446 men (age: *M* = 38.14, SD = 11.66, range: 18–80). We used an adapted version of the Kinsey Scale to assess sexual orientation (e.g., Kinsey et al., 1948, 1953). Details of the scale and descriptive data are presented in Supplement 4.

In Study 3, we collected data from a new sample to develop a short-form Hungarian YSEX questionnaire. This differs from the methodology described by Meston et al. (2019) in the development of the American version of the YSEX?-SF which used the original YSEX? data sample for item selection. The primary consideration underlying the development of the short form of the YSEX?-H questionnaire was that its internal consistency should not fall behind that of the full version (i.e., the Cronbach’s *α* coefficient for each factor and subfactor should be at least 0.60 that is an acceptable cut-off point for scales measuring broad concepts; Hoekstra et al., 2019). To this end, three short forms including 5, 4, and 3 items per subfactor were tested. The general rule of item selection was that the three items having the highest factor loadings within each subfactor should be included in the short form. Since the 3-item subfactors showed adequate internal consistency, these subfactors were included in the short form with one exception. Since the 3-item *Submission* subfactor of the *Sex as Coping* factor did not reach the expected Cronbach’s *α* value, all 4 items of this subfactor were included in the short form in order to obtain adequate internal consistency.

The only exception to this rule (apart from the above-mentioned *Submission* subfactor) was the *Dealing with Partner’s Emotional Needs* subfactor of the *Sex as Coping* factor. Items of this subfactor in descending order by factor loading were as follows: “It was a favor,” “Out of sympathy,” “Out of gratitude,” “Out of pity,” and “I wanted to comfort the other person.” However, a subfactor with more consistent contents was obtained when removing the items referring to sympathy and gratitude than when removing the last two items. All factors and subfactors of the resulting short form showed the expected internal consistency (see Table 6).

**Table 6:** Internal consistency of the long version (144 items) and short version (73 items) of the questionnaire and correlations of corresponding scales of the two versions (n = 1161)

Factor	Subfactor	Long version (144 items) Cronbach alpha	Short version (73 items) Cronbach alpha	*Pearson r*
Personal goal attainment		.95	.81	.98**
	Novelty seeking	.91	.74	.90**
	Conformity	.78	.76	.91**
	Infidelity	.84	.86	.91**
	Impulsiveness	.77	.75	.95**
	Revenge	.76	.75	.97**
	Sensation seeking	.83	.75	.93**
	Control and power	.67	.67	.97**
	Self-esteem boost	.85	.74	.95**
Relational reasons		.96	.88	.99**
	Sexual desire	.93	.86	.92**
	Commitment	.86	.79	.88**
	Physical attraction	.88	.74	.93**
	Relaxation	.82	.81	.97**
	Intimacy	.77	.68	.93**
	Excitement	.81	.72	.90**
	Self-affirmation	.79	.64	.92**
	Care	.72	.72	1,00***
	Happiness seeking	.66	.65	.93**
Sex as coping		.94	.81	.98**
	Emotional need satisfaction	.90	.78	.90**
	Compulsion and avoidance	.73	.66	.97**
	Utilitarianism	.71	.62	.80**
	Coping with relational conflicts	.81	.75	.95**
	Submission	.62	.62	.96**
	Dealing with partner’s emotional needs	.72	.65	.92**
	Mate retention	.86	.85	.96**

** *p* < .001, *** There were only 3 items in the long version, so the short version is the same

The item selection procedure used in the present study was similar to that used by Meston et al. (2019) which also relied on factor loadings as a basis for item selection in the development of the American short-form version of the scale. Our methodology differs from Meston et al., however, in that we selected the three highest loading items from each of the 24 subfactors (4 items from the *Submission* subfactor) resulting in a total of 73 items for the YSEX?-HSF; Meston et al. retained the two highest loading items from each of the 13 subfactors of the full scale YSEX? but made exceptions to select items with lower factor loadings for items that were not considered representative of the overall subscale and changed the wording of some items to be more broadly representative of the construct. Also, two items from an existing subfactor were used to form the basis of a new subscale. The resulting American YSEX?-SF consists of 28 items. It should be noted that the American YSEX?-SF was not published at the time the current study was conducted.

*Validation procedure* Following the validation procedure used by Meston and Buss (2007), the Sociosexual Orientation Inventory (SOI-R; Penke & Asendorpf, 2008; Hungarian version: Meskó et al., 2014) was administered to respondents in addition to the YSEX?-H questionnaire. Based on the Sexual Strategies Theory (Buss & Schmitt, 1993), we hypothesized that sociosexuality (i.e., openness to having sex in uncommitted relationships) could be associated with sexual motivations. Respondents also completed the 15-item GSOEP Big Five Inventory (BFI-S; Hahn et al., 2012). Based on the association of motivation in general and personality (e.g., Strus & Cieciuch, 2017), we hypothesized that factors of personality could be associated with sexual motivation. Since the BFI-S has not yet been adapted to Hungarian to our knowledge, we prepared a Hungarian translation for the purposes of the present study. Because of well-documented evidence for the cross-cultural invariance of Big Five dimensions (e.g., Salgado et al., 2003), we deemed cultural adaptation of BFI-S unnecessary.

#### Results

*Internal consistency* Internal consistency of the scales used in the study was tested by calculating Cronbach’s *α* coefficients with the SPSS v23 software. The coefficients obtained for the factors and subfactors of the YSEX?-H are shown in Table 7. The coefficients obtained for the SOI-R were as follows: Global Sociosexual Orientation: 0.86, Sociosexual Behavior: 0.78, Sociosexual Attitude: 0.84, Sociosexual Desire: 0.88. The coefficients obtained for the BFI-S were as follows: Extraversion: 0.69, Neuroticism: 0.73, Agreeableness: 0.44, Conscientiousness: 0.65, Openness: 0.54. According to Gosling et al. (2003), given the brevity of the BFI-S (i.e., three items per scale) and the breadth of the measured dimensions, these Cronbach’s α coefficients should raise no concern.

**Table 7:** Internal consistency of the 73 item YSEX?-HSF factor and subfactor items used in Study 3 (*n* = 1024)

Factor	Subfactor	Cronbach-alpha
Personal goal attainment		.88
	Novelty seeking	.72
	Conformity	.59
	Infidelity	.77
	Impulsiveness	.79
	Revenge	.75
	Sensation seeking	.69
	Control and power	.79
	Self-esteem boost	.75
Relational reasons		.92
	Sexual desire	.81
	Commitment	.73
	Physical attraction	.66
	Relaxation	.75
	Intimacy	.67
	Excitement	.69
	Self-affirmation	.53
	Care	.62
	Happiness seeking	.63
Sex as coping		.88
	Mitigating emotional deficit	.75
	Compulsion and avoidance	.61
	Utilitarianism	.72
	Coping with relational conflicts	.78
	Submissiveness	.57
	Coping with Partner’s emotional Demands	.50
	Mate retention	.82

Internal consistency scores are Cronbach’s coefficient alphas

*Confirmatory factor analysis of the short version* Confirmatory factor analyses were conducted with the AMOS add-on module for the SPSS v18.0 software in order to corroborate the reliability of the short form. Men’s and women’s responses were separately analyzed to verify that the reliability of the factor structure was not due to the predominance of women in the sample. The fit between the theoretical model predicting three related factors (see Appendix 1) and the data was tested by the RMSEA index, since the χ^2^ test was not informative due to the large sample size, while the relative fit indices were inadequate because the RMSEA of the null model did not reach the critical value of 0.158 (Kenny, 2015). The theoretical model showed acceptable fit both with women’s responses (*n* = 578; RMSEA = 0.045 [90% CI = 0.044–0.046]) and with men’s responses *(n* = 446; RMSEA = 0.058 [90% CI = 0.056–0.060]; Hu & Bentler, 1999). These results confirm that the short form of the YSEX?-H provides a reliable measure of the three distinct types of sexual motivation (i.e. Personal Goal Attainment, Relational Reasons, and Sex as Coping) both in women and men.

*The relationship between personality and sexual motivation in each gender group* Pearson’s correlation coefficients were calculated to test the relationship between the factors of the Big Five Inventory (BFI-S) and the factors and subfactors of the YSEX?-HSF in each gender group. The results are shown in Table 8. In sum, the factors and subfactors measuring sexual motivation positively correlated with Extraversion, Neuroticism and Openness, while they predominantly showed negative correlations with Agreeableness and Conscientiousness. Extraversion positively correlated with all three factors of the YSEX?-HSF (*Personal Goal Attainment*, *Relational Reasons*, and *Sex as Coping*) and showed the highest total number of significant correlations with sexual motives. Neuroticism showed a significant (positive) correlation with only one factor in the sample of women: those reporting higher levels of emotional instability scored higher on the *Sex as Coping* factor. Agreeableness correlated significantly (negatively) with only one factor: those who reported higher levels of Agreeableness scored higher on the *Personal Goal Attainment* factor. Conscientiousness was also associated (negatively) with only one factor in the male subsample: those judging themselves as more conscientious scored lower on the *Personal Goal Attainment* factor. Openness was positively associated with all three factors of the YSEX?-HSF. The subfactors of sexual motives outlined an even more complex picture (see Table 8).

**Table 8:** Pearson’s correlations between the 73-item YSEX?-HSF questionnaire and the Big Five Inventory

Factor/subfactor	Men					Women				
	E	N	A	C	O	E	N	A	C	O
Personal goal attainment	.22**	.02	− .09	− .09*	.15**	.11**	.07	− .14**	.02	.10*
Novelty seeking	.19**	− .04	− .06	− .05	.13**	.15**	− .05	− .09*	.08*	.10*
Conformity	.07	− .00	− .01	− .09*	.04	− .01	.06	− .04	.05	.01
Infidelity	.10*	.04	− .03	− .00	.09*	.04	− .04	− .08*	.14**	.05
Impulsiveness	.18**	.03	− .13**	− .15**	.07	.06	.11**	− .17**	− .15**	.04
Revenge	.11*	− .05	− .05	− .00	.08	− .02	.06	− .12**	.10**	.08
Sensation seeking	.18**	− .02	− .05	− .00	.14**	.15**	.01	− .12**	.03	.10*
Control and power	.16**	.07	− .12**	− .10*	.09	.05	.07	− .09*	.03	.08*
Self-esteem boost	.16**	.08	− .01	− .09*	.13**	.08	.12**	.00	− .05	.04
Relational reasons	.16**	− .03	.03	.03	.23**	.17**	.06	.02	.07	.11**
Sexual desire	.11*	.02	− .01	.03	.13**	.14**	.05	− .02	.05	.03
Commitment	.12*	− .09*	.01	.07	.09*	.11**	.00	.07	.08*	.06
Physical attraction	.10*	− .08	− .02	− .02	.19**	.10*	.06	− .03	.04	.13**
Relaxation	.10*	.07	.00	− .01	.16**	.15**	.01	− .02	− .01	.07
Intimacy	.18**	− .08	.10*	.14**	.25**	.16**	.03	.09*	.11**	.10*
Excitement	.12**	− .04	.04	− .02	.21**	.10**	− .05	− .05	.09*	.12**
Self-affirmation	.10*	.01	.02	− .03	.22**	.08*	.10*	− .01	.01	.10*
Care	.11*	.00	− .02	− .03	.11*	.03	.08	.00	− .04	.01
Happiness seeking	.10**	− .02	.11*	.08	.16**	.13**	.08*	.08	.05	.06
Sex as coping	.11*	.02	− .03	− .06	.13**	.04	.20**	− .07	− .08	.00
Emotional need Satisfaction	.05	.12**	− .05	− .09*	.11*	.03	.24**	− .08	− .09*	.03
Compulsion and avoidance	.01	− .02	− .01	− .05	.06	− .05	.07	− .08	− .05	− .05
Utilitarianism	.15**	− .02	− .00	− .02	.02	.00	.07	− .11**	.02	.04
Coping with relational conflicts	.14**	− .04	− .00	− .03	.13**	.09*	.12**	− .06	.01	− .02
Submission	.02	.07	− .02	− .03	.07	.05	.16**	.02	− .09*	.05
Dealing with partner’s emotional needs	.05	− .01	− .04	− .07	.06	− .03	.08*	− .01	− .03	− .02
Mate retention	.11*	− .03	− .00	.01	.12**	.05	.16**	− .04	− .08*	− .01

Labels: E = extraversion, N = neuroticism, A = agreeableness, C = conscientiousness, O = openness

*p < .05, **p < .001

The correlations between sexual motives and personality factors showed rather different patterns in the two gender groups. While Extraversion positively correlated with all the three factors in the male subsample, it was only associated with *Personal Goal Attainment* and *Relational Reasons* but not with *Sex as Coping* (nor with any of its subfactors) in the sample of women. Neuroticism only correlated (positively) with *Sex as Coping* among the three factors of the YSEX?-HSF in the sample of women: those women who judged themselves as more unstable reported to be more motivated by reasons related to coping (e.g., Mate Retention, Dealing With Partner’s Emotional Needs, Submission, Coping With Relational Conflicts). Agreeableness was not associated with any of the three factors of the YSEX?-HSF in the sample of men, but showed a negative correlation with *Personal Goal Attainment* in the sample of women. Conscientiousness only correlated (negatively) with the *Personal Goal Attainment* factor in the sample of men. Openness showed positive correlations with all three factors in the sample of men, but was only associated with *Relational Reasons* in the sample of women.

*Sociosexuality* The associations between the global sociosexual orientation index and various sexual motives were tested by calculating Pearson’s correlation coefficients for each gender group (see Table 9). Table 9 shows that the same general pattern was obtained for both men and women (positive correlations were found in all cases), but while almost all factors and subfactors of the YSEX?-HSF showed a marked association with sociosexuality in the sample of men, the same held true for only one subfactor of the *Sex as Coping* factor in the sample of women. Sociosexuality showed the only negative correlation with the *Intimacy* subfactor in both gender groups.

**Table 9:** Correlations between YSEX?-HSF factors and subfactors and revised sociosexual orientation inventory (SOI-R)

Factor and subfactor	Men	Women
Personal goal attainment	.53**	.51**
Novelty seeking	.35**	.35**
Conformity	.22**	.07
Infidelity	.26**	.17**
Impulsiveness	.21**	.26**
Revenge	.13**	.15**
Sensation seeking	.33**	.33**
Control and power	.22**	.17**
Self-esteem boost	.29**	.18**
Relational reasons	.12**	.02
Sexual desire	.24**	.11**
Commitment	.01	− .06
Physical attraction	.16**	.13**
Relaxation	.22**	.12**
Intimacy	− .10*	− .16**
Excitement	.22**	.09*
Self-affirmation	.05	.04
Care	.12**	− .04
Happiness seeking	.07	.00
Sex as coping	.20**	.10**
Mitigating emotional deficit	.24**	.21**
Compulsion and avoidance	.18**	.08*
Utilitarianism	.08	.06
Coping with relational conflicts	.09*	.04
Submissiveness	.15**	.07
Coping with partner’s emotional demands	.14**	.05
Mate retention	.07	.05

*p < .05, **p < .001

*The relationship between age and the factors and subfactors of the YSEX?-HSF* Spearman’s rank correlation coefficients were calculated to test the associations between age and the factors and subfactors of the 73-item YSEX-HSF questionnaire in both subsamples and in the overall sample. Significance was tested at two levels (*p* < 0.05 and *p* < 0.001; see Table 10). In sum, the findings obtained with the short form replicated those obtained with the full version (shown in Table 10). The direction and strength of significant correlations were similar between questionnaires. The three factors of the YSEX-HSF and YSEX-H also showed corresponding correlations: age did not correlate with *Personal Goal Attainment* in either case, but showed a significant negative correlation with *Relational Reasons* and with *Sex as Coping* in both cases.

**Table 10:** Spearman’s rank correlations (ρ) between age and the factors and subfactors of the YSEX?-HSF questionnaire

Factor and subfactor (73 items)	rho		
	Overall (*n* = 1024)	Women (*n* = 578)	Men (*n* = 446)
Personal goal attainment	.02	− .02	− .05
Novelty seeking	.07	− .01	.06
Conformity	.44	.07	− .08
Infidelity	.27**	.17**	.26**
Impulsiveness	− .16**	.09*	− .25**
Revenge	− .33	.04	− .07
Sensation seeking	.16	− .30	− .05
Control and power	− .20	.02	− .09
Self-esteem boost	− .67*	− .02	− .10*
Relational reasons	− .14**	− .10*	− .14**
Sexual desire	− .14	− .03	.01
Commitment	− .12**	− .02	− .18**
Physical attraction	.01	− .02	− .03
Relaxation	− .14**	− .08	− .19**
Intimacy	− .14**	− .06	− .11*
Excitement	− .09**	− .12**	− .12*
Self-affirmation	− .12**	− .07	− .60
Care	− .25**	− .24**	− .23**
Happiness seeking	− .04	− .02	− .01
Sex as coping	− .13**	− .10*	− .05
Emotional need satisfaction	− .13**	− .06	− .11*
Compulsion and avoidance	− .01	− .01	.06
Utilitarianism	− .11**	− .07	− .11
Coping with relational conflicts	− .15**	− .13**	− .12*
Submission	− .15**	− .07	− .10*
Dealing with partner’s emotional needs	− .02	− .02	.01
Mate retention	− .05	− .01	.01

*p < .05, **p < .01

## Discussion

To our knowledge, the Hungarian adaptation of the YSEX? questionnaire (Meston & Buss, 2007) is the first adaptation based on population-specific data rather than on a translation of the original questionnaire. The primary aim of the present studies was to develop a questionnaire that tapped into the diversity of sexual motives in the Hungarian population and followed the exact methodology used by Meston and Buss (2007).^2^ Our findings expand existing knowledge of human sexuality in several respects and contribute to a better understanding of the complexity of sexual motivation. In comparison with the original questionnaire, (1) a different structure was found (while most items were similar); (2) marked gender differences were found in the characteristic patterns of sexual motives; (3) an age effect on sexual motivation was revealed; (4) a short (73-item) form of the questionnaire was developed. These findings are discussed below in detail.

### Differences and Similarities Between the Original YSEX? and the YSEX?-H Questionnaires

While the original YSEX? questionnaire (Meston & Buss, 2007) was based on 237 reasons for having sex, development of the YSEX?-H initially revealed 197 distinct reasons (see Study 1). The original YSEX? and the Hungarian adaptation have many items with overlapping or identical content (e.g., “I was seeking experience,” “I was under the influence of alcohol,” “I was in love”), while other items are only included in one or the other questionnaire (e.g., “I wanted to give someone else a sexually transmitted disease,” “The person was intelligent,” “I wanted to feel feminine/masculine”). The difference between the two questionnaires in the number of items is mainly due to items that describe age and culture-specific reasons characteristic to young adults (university students), which reasons are generally related to the psychological problems of the transition from adolescence to adulthood (e.g., “I wanted to feel older,” ”I wanted to defy my parents”).

### Differences and Similarities in the Factor Structures

The original YSEX? and the YSEX?-H show a striking difference in the number of factors (4 vs. 3). A certain shift of focus is observable in the three-factor structure of the YSEX?-H (Personal Goal Attainment, Relational Reasons, Sex as Coping) as compared to the four-factor structure of the original YSEX? (Physical Reasons, Goal Attainment, Insecurity, Emotional Reasons) developed by Meston and Buss (2007). Although the two questionnaires list very similar reasons, these reasons seem to be articulated in different ways when examined in the context of the factor structures. The *Personal Goal Attainment* factor of the YSEX?-H partly overlaps the *Physical Reasons* and *Goal Attainment* factors and their subfactors in the original YSEX? questionnaire. For example, an item referring to experimentation is present in both *Personal Goal Attainment* and *Physical Reasons* factors of the two questionnaires (“I wanted to experiment with new experiences” and “I wanted to seek experience,” for YSEX? and YSEX?-H, respectively). At the same time, an item referring to revenge as a motivation for having sex is to be found in both *Personal Goal Attainment* and *Goal Attainment* factors of the two questionnaires (“I wanted to get back at my partner for having cheated on me” and “I wanted to take revenge,” for YSEX? and YSEX?-H, respectively). Furthermore, the *Relational Reasons* and *Sex as Coping* factors of the YSEX?-H are very similar to the *Emotional Reasons* and *Insecurity* factors of the YSEX? questionnaire, respectively. “I realized I was in love” (YSEX?) and “I was in love” (YSEX?-H) are from *Emotional Reasons* and *Relational Reasons* factors, respectively. Meanwhile, “I wanted to say «I’m sorry»” (YSEX?) and “I wanted to apologize.” (YSEX?-H) are from *Insecurity* and *Sex as Coping* factors, respectively.

These comparisons raise the question of whether the observed differences and similarities are due to cultural factors. In our view, the difference between the factor structures is most likely a result of age differences between the samples. A Norwegian study successfully replicated the factor structure of the original YSEX? questionnaire despite the cultural differences between the Norwegian and American respondents (Kennair et al., 2015). However, the Norwegian sample and the American sample both included university students.

### Gender Differences and Similarities in Sexual Motivation

The present study revealed robust gender differences in the characteristic patterns of various sexual motives. The observed similarities and differences between men’s and women’s sexual motivation are discussed below.

1. *Similarities* Men and women showed more similarities than differences in the most important reasons for having sex (e.g., love, mutual attraction, sexual desire). However, biological sex influences which specific attributes of potential partners elicit the experience of love in men and women (Hendrick et al., 1984; Sprecher & Toro-Morn, 2002). From an evolutionary point of view, this means that gender similarities in sexual motivation are underpinned by aspirations that have sex-specific origins and functions. Thus, the function of romantic or erotic love may be the same for each gender (e.g., strengthening the emotional attachment of a relationship and a sense of belonging), but it is worth clarifying that women and men are triggered by other factors (e.g., Conroy-Beam et al., 2019; Meskó et al., 2021; Schmitt, 2014; Walter et al., 2020). For example, women are more likely to experience love and attraction to dominant high-status men, while men are more likely to feel love and attraction toward physically attractive women (Fletcher et al., 2004). Women tend to associate love with emotional attachment and security, while men are more likely to define love in terms of sexual fidelity and satisfaction (Buss, 2000). This was corroborated by the present study, which found no significant difference between men and women on the *Relational Reasons* factor (e.g., Excitement, Care, Relaxation, Sexual Desire).
2. *Differences* The present study revealed a number of marked gender differences in the characteristic patterns of sexual motives. Men primarily attributed their sexual activity to *Personal Goal Attainment* (e.g., Novelty Seeking, Impulsiveness, Infidelity), while women assigned more importance to *Sex as Coping* (e.g., Submission, Mate Retention). Gender differences in sexual motivations can be explained by several theoretical perspectives. According to social scripting theory (e.g., Gagnon, 1990; Wiederman, 2005), in Western cultures, scripts for sexual activity are markedly different for men and women. Social sexual scripts can be seen as a type of social agents that prescribe what counts as normative within a culture, thus providing guidance on how to feel, think, and behave in a given situation. Wiederman (2005) argues that the higher number of casual sexual partners reported by men—as compared to women—in self-reported questionnaires is the result of social sexual scripts. In other words, masculinity expectations position having many sexual partners as an achievement for men. Among the psychological mechanisms underlying gender differences in openness to casual sex, Conley (2011) highlights the importance of pleasure.

According to the pleasure theory (Abramson & Pinkerton, 2002), the pursuit of sexual pleasure is the central basis of human sexual motivation (and not the desire to reproduce). In this context, Conley (2011) emphasized that casual sexual contact with a man is likely to be less enjoyable for a woman than vice versa. Interpersonal factors directly related to the relationship may also have an effect on women and men reporting different sexual motivations in research. According to the literature on sexual coercion (e.g., Livingston et al., 2004; Struckman‐Johnson, 1988) women—as compared to men—are more likely to have partners who threaten to break up with them if they do not have sex. In addition to proximal (direct) explanations describing the effects of social variables on sexual behavior and motivations, we should also address possible ultimate (evolutionary) explanations for gender differences.

A plausible explanation for the gender differences in various sexual motives and mate preferences is offered by the *parental investment theory* proposed by Trivers (1972). In the animal kingdom, females and males make unequal contributions to parental investment expressed in the time and energy allocated to each offspring. Individuals of the sex investing less time and energy in parenting allocate more resources to mating with individuals of the opposite sex. The former sex is more aggressive and reaches maturity later than the opposite sex, while it has a lower life expectancy, and its behavior is generally more energetic, active, and dynamic. This sex is less selective in mating: it aims to access as many mates as possible by investing as little time and energy as possible. In most species, this strategy is followed by males. Females invest more in producing offspring, which is especially true for species with internal fertilization such as mammals. This model is clearly consistent with the finding that men are more likely to engage in short-term non-committed sexual relationships than women. Trivers’ theory has been supported by several empirical studies (e.g., Buss & Schmitt, 1993). As was the case in the present study, other studies also found greater gender differences in sexual motivation than those obtained by Meston and Buss (Gouvernet et al., 2017; Kennair et al., 2015; Meston & Buss, 2007).

### The Impact of Age on Sexual Motivation

The present study found that older people generally scored lower on sexual motives than did younger people, which suggests the importance and function of sexual activity in an intimate partner relationship may change over time. Keeping in mind that desire to engage in sex is only one category of sexual motivation, the finding of a decline in sexual motivation in older versus younger persons is consistent with the well-documented decline in sexual desire seen with age in both men and women (e.g., Hatfield & Rapson, 2015; Höglund et al., 2014; Loe, 2012), and with the related decline in testosterone with age (Dick et al., 2020; Krapf & Simon, 2017; Maggi et al., 2020). However, it should be noted that the relationship between testosterone and sexual desire is not yet completely clear (e.g., van Anders, 2012). The finding of a decrease in sexual motives with age is in striking contrast with the finding on the importance of infidelity at different ages. While younger people reported being primarily responsive to Physical Attraction, Intimacy and Care, that is, to *Relational Reasons*, older people were more motivated by *Personal Goal Attainment* (e.g., Infidelity), which may be considered a manifestation of their desire for sexual variety. These findings are in line with those reported by Wyverkens et al. (2018), who administered the original YSEX? questionnaire to an older sample and drew similar conclusions.

### Personality and Sexual Motivation

The factors of the YSEX?-HSF showed a more complex pattern of associations with the Big Five personality factors than that seen in the original U.S. study (Meston & Buss, 2007). The present study revealed a positive relationship between Extraversion and sexual motivation, which is consistent with the findings of several previous studies (e.g., Gangestad & Simpson, 2000; Jonason et al., 2016; Schmitt, 2004). Neuroticism and sexual motivation also showed a positive association (among women), which corroborates findings of previous studies (Allen & Walter, 2018; Pinkerton & Abramson, 1996; Trobst et al., 2002). Agreeableness and Conscientiousness were negatively associated with the Personal Goal Attainment subfactors of sexual motivation. This finding is highly similar to that reported by Schmitt (2004), who found that sexual promiscuity and infidelity was universally associated with low Agreeableness and low Conscientiousness. All three subfactors of the YSEX?-HSF showed a positive relationship with Openness, which is in line with the finding reported by Schmitt and Shackelford (2008), who revealed a positive association between Openness and short-term mating. Another related finding was reported by Moyano and Sierra (2013), who revealed that openness to experience was negatively associated with negative sexual cognitions. The associations revealed between the YSEX?-HSF and the Big Five personality factors are highly consistent with previous findings on the relationship between sexual motivation and personality.

As compared to the original YSEX? questionnaire, the YSEX?-HSF revealed more pronounced associations between sexual motivation and personality. This may be due to differences between studies in sample characteristics, with the Hungarian studies including older, more mature adults as opposed to university students.

## Conclusions

To our knowledge, the YSEX?-HSF is the first national adaptation of the YSEX? questionnaire whose development was based on motives reported by a national sample as opposed to a translation of the original questionnaire. The YSEX?-HSF is a reliable measure with good predictive validity, which confirmed the major expected associations of sexual motivation with both sociosexuality and the Big Five personality factors. Furthermore, the YSEX?-HSF revealed marked gender and age differences in sexual motivation. The YSEX?-HSF showed a factor structure different from that of the original American version of the questionnaire: the former is composed of three primary factors comprising 24 subfactors; the latter consists of four primary factors and 13 subfactors. Despite the differences between factor structures, the composition of reasons for having sex is highly similar in the two factors. This finding points to both the cross-cultural universality of human sexual motivation, and also the cultural diversity reflected in the differences between the two factor structures. The YSEX?-HSF questionnaire is presented in Supplement 5.

There are a number of study limitations that warrant mention. First, the self-report methodology used in the present studies was based on the assumption that respondents had conscious access to their sexual motives, which is not necessarily true in all cases. Second, data obtained with self-report measures might be distorted by social desirability effects: respondents might suppress socially undesirable responses (e.g., having sex with someone to punish the partner, giving sex for money, etc.), whereas they might show a bias toward socially desirable responses (e.g., having sexual contact to express positive emotions toward the partner, being driven by love, etc.). Furthermore, respondents might be motivated to present themselves in accordance with gender stereotypes. As a consequence, men might have chosen responses that depicted them as sexually more active, more potent or more masculine than they actually were, while women might have suppressed these same responses. Third, although the samples used in the studies were large and relatively heterogeneous, they were not necessarily representative. For example, the samples may not have included asexual respondents, who are likely to be uninterested in a study on sexual motivation.

## Supplementary Information

Below is the link to the electronic supplementary material.

Supplementary file1 (DOCX 44 kb)

Supplementary file2 (DOCX 16 kb)

Supplementary file3 (DOCX 18 kb)

Supplementary file4 (DOCX 14 kb)

Supplementary file5 (DOCX 25 kb)

## Footnotes

1 The first primary factor is *Physical reasons*, which comprises four subfactors: *Stress Reduction* (e.g., “I thought it would relax me”), *Pleasure* (e.g., “I wanted to experience the physical pleasure”), *Physical Desirability* (e.g., “The person’s physical appearance turned me on”), and *Experience Seeking* (e.g., “I was curious about sex”). The second primary factor is *Goal Attainment Reasons*, which also included four subfactors: *Resources* (e.g., “I wanted to get a job”), *Social Status* (e.g., “I wanted to be popular”), *Revenge* (e.g., “I wanted to make someone else jealous”), and *Utilitarian* (e.g., “I wanted to burn calories”). The third primary factor is *Emotional Reasons*, which included two subfactors: one is *Love and Commitment* (e.g., “I wanted to become one with another person”), the other is *Expression* (e.g., “I wanted to say “I’m sorry”). The fourth primary factor is *Insecurity Reasons* comprising three subfactors*: Self-Esteem Boost* (e.g., “I wanted to feel powerful”), *Duty/Pressure* (e.g., “I didn’t want to disappoint the person”), and *Mate Guarding* (e.g., “I wanted to prevent a breakup”). The study involved 1549 university students (mostly aged 18 to 22). The specificity of this sample possibly restricts the validity of the findings, since it is uncertain whether the questionnaire may be used in its original form to assess older age groups’ sexual motivation.

2 Items of YSEX?-H are available in Table 1. The full formatted version of the questionnaire is available from the corresponding author.
