# Supplementary material for: Why Hungarians Have Sex (YSEX?-HSF)
Source: Arch Sex Behav. 2021 Nov 12;51(1):465–89. doi: 10.1007/s10508-021-02072-y (PMC8858278; doi:10.1007/s10508-021-02072-y)
Supplement: Supplementary file 1 — Supplementary file1 (DOCX 44 kb) [file 10508_2021_2072_MOESM1_ESM.docx]

Supplement 1

*The most and least frequent reasons for having sexual intercourse (1/A and 1/B), and the gender differences in the causes leading to intercourse (1/C)*

The most frequent reasons leading respondents to have sexual intercourse were as follows: (1) I was in love, (2) I enjoy having sex, (3) out of hedonism, (4) out of mutual attraction, (5) to please myself, (6) to feel happy, (7) to satisfy my partner, (8) out of sexual desire. Table 1 shows which 50 of the 197 reasons had the highest importance in having sexual contact (intercourse) within each gender group. Table 1 reveals that men’s and women’s most important reasons largely overlapped: they had 8 of the top 10 reasons and 22 of the top 25 reasons in common.

**Supplement 1/A**

*Top 50 reasons why men and women have sex*

| Item | Women | |  | Item | Men | |
| --- | --- | --- | --- | --- | --- | --- |
|  | M | SD |  |  | M | SD |
| 1. I was in love. | 4.13 | 1.40 |  | I love sex. | 4.01 | 1.24 |
| 1. Out of mutual appeal. | 3.90 | 1.18 |  | To seek pleasure. | 3.98 | 1.20 |
| 1. To seek pleasure. | 3.89 | 1.25 |  | I was in love. | 3.95 | 1.18 |
| 1. I wanted to make my partner happy. | 3.84 | 1.22 |  | Because of sexual desire. | 3.91 | 1.23 |
| 1. Because of sexual desire. | 3.83 | 1.23 |  | I wanted to satisfy my partner. | 3.87 | 1.28 |
| 1. I wanted to please myself. | 3.79 | 1.26 |  | I wanted to please myself. | 3.87 | 1.22 |
| 1. I love sex. | 3.78 | 1.30 |  | Out of mutual appeal. | 3.76 | 1.20 |
| 1. Out of passion. | 3.70 | 1.27 |  | I wanted to be/feel happy. | 3.65 | 1.35 |
| 1. I wanted to satisfy my partner. | 3.65 | 1.27 |  | The person was a desirable/sexy. | 3.59 | 1.20 |
| 1. I wanted to be/feel happy. | 3.64 | 1.34 |  | I wanted pleasure. | 3.57 | 1.25 |
| 1. The touch of the person was pleasant. | 3.58 | 1.31 |  | Out of affection. | 3.48 | 1.34 |
| 1. The person was a desirable/sexy. | 3.55 | 1.27 |  | I wanted to have an orgasm. | 3.47 | 1.35 |
| 1. Out of affection. | 3.53 | 1.36 |  | The touch of the person was pleasant. | 3.47 | 1.36 |
| 1. I wanted pleasure. | 3.49 | 1.30 |  | Out of passion. | 3.45 | 1.34 |
| 1. I wanted to be satisfied. | 3.45 | 1.34 |  | I wanted to be satisfied. | 3.35 | 1.38 |
| 1. I wanted to have an orgasm. | 3.44 | 1.37 |  | I was horny. | 3.26 | 1.33 |
| 1. I was horny. | 3.38 | 1.37 |  | The person had an attractive body. | 3.12 | 1.27 |
| 1. I wanted to become one with the other person. | 3.27 | 1.41 |  | The person was too ‘‘hot’’ (sexy) to resist. | 3.08 | 1.29 |
| 1. It was a way to express my feelings. | 3.22 | 1.41 |  | I wanted to become one with the other person. | 3.07 | 1.42 |
| 1. I wanted the relationship to grow. | 3.21 | 1.41 |  | It was a way to express my feelings. | 3.06 | 1.40 |
| 1. I wanted to be attached to the person. | 3.20 | 1.40 |  | I wanted the relationship to grow. | 3.04 | 1.42 |
| 1. We were made for each other. | 3.12 | 1.43 |  | We were made for each other. | 2.99 | 1.40 |
| 1. I was in a romantic mood. | 3.10 | 1.23 |  | I was in a romantic mood. | 2.94 | 1.22 |
| 1. The person was too ‘‘hot’’ (sexy) to resist. | 3.09 | 1.34 |  | I needed it. | 2.93 | 1.33 |
| 1. I wanted to deepen the relationship. | 3.07 | 1.37 |  | It was a pleasant pastime. | 2.92 | 1.33 |
| 1. Out of trust. | 3.03 | 1.54 |  | I wanted to seek experience. | 2.92 | 1.27 |
| 1. It was a pleasant pastime. | 2.99 | 1.36 |  | The person had an attractive personality. | 2.90 | 1.28 |
| 1. I needed it. | 2.91 | 1.33 |  | I wanted to deepen the relationship. | 2.87 | 1.36 |
| 1. The person had an attractive personality. | 2.90 | 1.32 |  | I wanted to be attached to the person. | 2.74 | 1.40 |
| 1. I wanted to intensify the commitment. | 2.84 | 1.38 |  | It seemed like the next step in the relationship. | 2.71 | 1.34 |
| 1. I wanted to get closer to the person. | 2.78 | 1.31 |  | It was a seduction/I was seduced. | 2.65 | 1.20 |
| 1. It seemed like the next step in the relationship. | 2.75 | 1.36 |  | I wanted to act out a fantasy. | 2.62 | 1.26 |
| 1. I wanted to seek experience. | 2.68 | 1.31 |  | I liked the person. | 2.60 | 1.29 |
| 1. It was a seduction/I was seduced. | 2.63 | 1.23 |  | I wanted to conquer the other person. | 2.60 | 1.30 |
| 1. I was seeking emotional closeness (i.e., intimacy). | 2.63 | 1.32 |  | Out of trust. | 2.59 | 1.47 |
| 1. To get on the same wavelength with the other person. | 2.62 | 1.36 |  | I wanted to get closer to the person. | 2.57 | 1.28 |
| 1. I wanted to recharge myself. | 2.60 | 1.35 |  | I took the opportunity. | 2.54 | 1.14 |
| 1. I wanted to conquer the other person. | 2.60 | 1.31 |  | I wanted to intensify the commitment. | 2.52 | 1.31 |
| 1. The person had an attractive body. | 2.59 | 1.33 |  | I was seeking emotional closeness (i.e., intimacy). | 2.49 | 1.29 |
| 1. I wanted to act out a fantasy. | 2.53 | 1.28 |  | I wanted to satisfy a deficit. | 2.47 | 1.25 |
| 1. Out of impulse. | 2.52 | 1.21 |  | I wanted a new experience. | 2.47 | 1.15 |
| 1. I liked the person. | 2.44 | 1.34 |  | Out of impulse. | 2.46 | 1.10 |
| 1. It was a special occasion/situation. | 2.42 | 1.18 |  | The person offered herself/himself. | 2.46 | 1.13 |
| 1. I wanted to have sex in an unusual place or situation. | 2.40 | 1.19 |  | I wanted to have sex in an unusual place or situation. | 2.45 | 1.16 |
| 1. It was an inner drive (I couldn’t control myself). | 2.37 | 1.33 |  | I love variety. | 2.44 | 1.35 |
| 1. The person smelled good. | 2.37 | 1.29 |  | It was a special occasion/situation. | 2.43 | 1.15 |
| 1. I wanted to satisfy a deficit. | 2.34 | 1.26 |  | To get on the same wavelength with the other person. | 2.42 | 1.30 |
| 1. I took the opportunity. | 2.30 | 1.21 |  | Out of curiosity. | 2.41 | 1.20 |
| 1. I wanted a new experience. | 2.27 | 1.15 |  | The person smelled good. | 2.39 | 1.27 |
| 1. Out of curiosity. | 2.27 | 1.16 |  | I wanted to recharge myself. | 2.39 | 1.28 |
| Note: Absolute range 1–5, *n* = 820 for women; *n* = 341 for men | | | | | | |

**Supplement 1/B**

*The 50 most infrequent reasons why men and women have sex*

Supplement 1/B shows which 50 of the 197 reasons had the lowest importance in having sexual contact (intercourse) within each gender group. Men’s and women’s least important reasons also largely overlapped (43 of the 50 reasons were common). It is difficult to consistently sort these reasons into a small number of meaningful categories, but generally 5 major themes seem to emerge. The first theme is social conformity or pressure (e.g. ‘I did not want to be odd man out’). The second theme is exerting pressure on the partner (e.g. ‘to have control over the partner’). The third theme is obtaining resources in return for sex (e.g. money, career progress, benefits). The fourth theme is engaging in sexual activity in response to threat (e.g. violence, blackmailing). The fifth theme is punishing the current or absent partner (e.g. ‘to humiliate the partner’; ‘to take revenge’).

| Item | Women | | |  | Item | Men | |
| --- | --- | --- | --- | --- | --- | --- | --- |
|  | M | SD |  |  | | M | SD |
| 1. For career advancement. | 1.02 | .16 |  | For livelihood. | | 1.02 | .13 |
| 1. Due to religious ritual. | 1.02 | .21 |  | Due to religious ritual. | | 1.02 | .16 |
| 1. For livelihood. | 1.03 | .25 |  | For career advancement. | | 1.02 | .19 |
| 1. Instead of paying or getting paid. | 1.03 | .26 |  | Due to violence. | | 1.04 | .27 |
| 1. Out of shame. | 1.03 | .23 |  | Due to a threat. | | 1.04 | .31 |
| 1. For money. | 1.05 | .31 |  | Out of shame. | | 1.05 | .24 |
| 1. Due to a threat. | 1.05 | .30 |  | For money. | | 1.05 | .33 |
| 1. For profit. | 1.05 | .33 |  | Instead of paying or getting paid. | | 1.05 | .30 |
| 1. I blackmailed him or her / He or she blackmailed me. | 1.06 | .33 |  | For profit. | | 1.06 | .38 |
| 1. Out of envy. | 1.06 | .35 |  | I blackmailed him or her / He or she blackmailed me. | | 1.07 | .35 |
| 1. Due to violence. | 1.07 | .34 |  | Out of fear. | | 1.07 | .37 |
| 1. I wanted to humiliate the person. | 1.08 | .35 |  | Out of envy. | | 1.08 | .44 |
| 1. I wanted to gain financial interest. | 1.08 | .42 |  | I wanted to gain financial interest. | | 1.10 | .44 |
| 1. I was a bet. | 1.09 | .38 |  | I wanted to benefit from it. | | 1.10 | .44 |
| 1. I wanted to benefit from it. | 1.10 | .44 |  | I wanted to satisfy my masochistic instincts. | | 1.11 | .42 |
| 1. It was trendy. | 1.11 | .43 |  | I was a bet. | | 1.13 | .40 |
| 1. I wanted to punish my partner. | 1.11 | .44 |  | I wanted to humiliate the person. | | 1.13 | .42 |
| 1. I was under the influence of drugs. | 1.11 | .44 |  | I hoped it would lead to advancement. | | 1.13 | .57 |
| 1. I hoped it would lead to advancement. | 1.12 | .53 |  | It was a rebellion against parental rules. | | 1.13 | .47 |
| 1. He/she offered me something in exchange for sex. | 1.12 | .50 |  | I wanted to punish a cheater. | | 1.13 | .46 |
| 1. I wanted to show off. | 1.13 | .50 |  | I was under the influence of drugs. | | 1.15 | .54 |
| 1. Out of fear. | 1.13 | .49 |  | I wanted to infuriate someone. | | 1.15 | .48 |
| 1. I wanted to increase my prestige. | 1.14 | .51 |  | It was trendy. | | 1.16 | .53 |
| 1. I was having midlife anxiety. | 1.15 | .55 |  | I wanted to punish my partner. | | 1.16 | .50 |
| 1. I wanted to infuriate someone. | 1.16 | .48 |  | I wanted to take revenge. | | 1.18 | .58 |
| 1. It was a favor. | 1.16 | .51 |  | I wanted to make my partner jealous. | | 1.18 | .53 |
| 1. I wanted to punish a cheater. | 1.16 | .52 |  | I wanted to experience aggression. | | 1.19 | .61 |
| 1. It was an initiation. | 1.17 | .54 |  | Due to rivalry (to show someone I'm better than a certain someone). | | 1.19 | .50 |
| 1. As a jape. | 1.17 | .53 |  | He/she offered me something in exchange for sex. | | 1.20 | .67 |
| 1. I wanted to be cool. | 1.17 | .56 |  | I wanted to show off. | | 1.21 | .54 |
| 1. I wanted to satisfy my masochistic instincts. | 1.18 | .58 |  | Out of compassion. | | 1.22 | .60 |
| 1. I didn’t want to be the odd one out. | 1.20 | .56 |  | I wanted to demonstrate my power. | | 1.23 | .67 |
| 1. Out of compassion. | 1.21 | .54 |  | Out of remorse. | | 1.24 | .63 |
| 1. Due to rivalry (to show someone I'm better than a certain someone). | 1.22 | .60 |  | As a jape. | | 1.25 | .64 |
| 1. It was a rebellion against parental rules. | 1.22 | .65 |  | It was a favor. | | 1.25 | .64 |
| 1. I wanted to take revenge. | 1.22 | .63 |  | I wanted to increase my prestige. | | 1.26 | .70 |
| 1. I wanted to demonstrate my power. | 1.22 | .65 |  | It was a way to reach my goal. | | 1.26 | .65 |
| 1. My partner was not able to have sex with me (so I had sex with someone else). | 1.23 | .66 |  | I didn’t want to be the odd one out. | | 1.26 | .67 |
| 1. I wanted to experience aggression. | 1.23 | .63 |  | It was an initiation. | | 1.27 | .69 |
| 1. I wanted to make my partner jealous. | 1.23 | .59 |  | I wanted to give in. | | 1.29 | .73 |
| 1. I wanted to record it (sound/picture/video). | 1.23 | .60 |  | I wanted to reduce anxiety. | | 1.30 | .72 |
| 1. It was a way to reach my goal. | 1.25 | .62 |  | I wanted to be cool. | | 1.30 | .72 |
| 1. My partner is not adventurous enough (so I had sex with someone else). | 1.28 | .75 |  | I wanted to submit myself. | | 1.31 | .76 |
| 1. My partner did not want to have sex with me (so I had sex with someone else). | 1.29 | .77 |  | I was afraid that my partner would leave me (if I didn’t have sex with him/her. | | 1.32 | .66 |
| 1. Out of remorse. | 1.30 | .68 |  | I wanted to decrease my anger. | | 1.32 | .74 |
| 1. I wanted to avert my partner’s suspicion. | 1.30 | .69 |  | I wanted to control the other person. | | 1.32 | .74 |
| 1. I wanted to reduce anxiety. | 1.31 | .70 |  | Out of despair. | | 1.33 | .71 |
| 1. I wanted to control the other person. | 1.31 | .71 |  | I wanted to influence the other person. | | 1.33 | .75 |
| 1. Out of gratitude. | 1.34 | .76 |  | I wanted to express my forgiveness this way. | | 1.34 | .65 |
| 1. I wanted to prove myself to others. | 1.34 | .78 |  | I was having midlife anxiety. | | 1.34 | .80 |
| *Note*: Absolute range. 1–5. n = 820 for women; n = 341 for men. | | | | | | | |

**Supplement 1/C**

*Gender differences in the causes leading to intercourse are ranked according to Cohen's d value, from the most characteristic of men, to the most characteristic of women.*

Item-level gender differences were analyzed by conducting 197 independent samples *t*-tests at a conservative significance level (*p* < .005). Of the 197 items, 64 (32%) showed significant gender differences. Men scored higher on two thirds (42) of these items and women on one third (22) of them. Detailed results of the statistical tests (*t* values, means, standard deviations, Cohen’s *d* coefficients) are shown in Supplement 1/C.

| Item | Women | | Men | | *t* | *d* |
| --- | --- | --- | --- | --- | --- | --- |
|  | M | SD | M | SD |  |  |
| The other person offered himself or herself. | 1.79 | 1.10 | 2.46 | 1.13 | 9.42 | -.60 |
| The person had an attractive body. | 2.37 | 1.29 | 3.12 | 1.27 | 9.09 | -.59 |
| The person had an exotic appearance. | 1.44 | .86 | 1.97 | 1.14 | 8.74 | -.53 |
| The other person was acting sexing. | 1.61 | .98 | 2.12 | 1.12 | 7.76 | -.49 |
| I was having midlife anxiety. | 1.07 | .36 | 1.34 | .81 | 7.77 | -.43 |
| My partner is not adventurous enough (so I had sex with someone else). | 1.18 | .62 | 1.52 | .96 | 7.35 | -.42 |
| Because I was addicted to sex. | 1.46 | .97 | 1.91 | 1.24 | 6.60 | -.40 |
| I wanted to prove myself to others. | 1.25 | .66 | 1.55 | .97 | 5.97 | -.36 |
| Out of friendship. | 1.43 | .80 | 1.74 | 1.05 | 5.61 | -.33 |
| For the hunt. | 1.66 | 1.05 | 2.03 | 1.19 | 5.22 | -.33 |
| I needed a one-night stand. | 1.78 | 1.04 | 2.14 | 1.19 | 5.15 | -.32 |
| I wanted the excitement of cheating on my partner. | 1.34 | .70 | 1.62 | 1.00 | 5.49 | -.32 |
| I love variety. | 2.03 | 1.21 | 2.44 | 1.35 | 5.08 | -.32 |
| My partner did not want to have sex with me (so I had sex with someone else). | 1.22 | .68 | 1.48 | .93 | 5.30 | -.32 |
| I wanted to increase my prestige. | 1.09 | .41 | 1.26 | .70 | 4.99 | -.30 |
| I wanted to be cool. | 1.12 | .47 | 1.3 | .72 | 5.12 | -.29 |
| I took the opportunity. | 2.2 | 1.22 | 2.54 | 1.14 | 4.41 | -.29 |
| I wanted to record it (sound/picture/video). | 1.18 | .49 | 1.36 | .80 | 4.83 | -.27 |
| It was forbidden. | 1.69 | 1.05 | 1.98 | 1.11 | 4.27 | -.27 |
| I wanted to seek experience. | 2.58 | 1.31 | 2.92 | 1.27 | 4.04 | -.26 |
| I love sex. | 3.68 | 1.32 | 4.01 | 1.24 | 3.94 | -.26 |
| I wanted to have a child. | 1.52 | 1.02 | 1.8 | 1.15 | 4.10 | -.26 |
| I wanted to discover the unknown. | 2.06 | 1.14 | 2.36 | 1.24 | 3.94 | -.25 |
| I wanted to show off. | 1.09 | .41 | 1.21 | .54 | 3.80 | -.25 |
| To prove myself. | 2.02 | 1.20 | 2.33 | 1.30 | 3.90 | -.25 |
| I wanted a new experience. | 2.19 | 1.14 | 2.47 | 1.15 | 3.75 | -.24 |
| I wanted to satisfy my partner. | 3.56 | 1.26 | 3.87 | 1.28 | 3.86 | -.24 |
| I wanted to make my partner happy. | 3.75 | 1.24 | 4.04 | 1.17 | 3.69 | -.24 |
| It was an initiation. | 1.13 | .46 | 1.27 | .69 | 4.04 | -.24 |
| I couldn’t say no. | 1.47 | .86 | 1.7 | 1.06 | 3.83 | -.24 |
| It was a favor. | 1.12 | .45 | 1.25 | .64 | 3.86 | -.24 |
| It was a challenge. | 1.66 | 1.01 | 1.91 | 1.14 | 3.70 | -.23 |
| My partner was not able to have sex with me (so I had sex with someone else). | 1.18 | .60 | 1.34 | .78 | 3.81 | -.23 |
| Out of infidelity. | 1.48 | .86 | 1.69 | .98 | 3.59 | -.23 |
| I wanted to try out new sexual techniques or positions. | 2.1 | 1.15 | 2.36 | 1.18 | 3.43 | -.22 |
| Instead of talking. | 1.45 | .77 | 1.64 | 1.02 | 3.49 | -.21 |
| I wanted to get the other person. | 1.97 | 1.16 | 2.22 | 1.30 | 3.23 | -.20 |
| As a jape. | 1.14 | .47 | 1.25 | .64 | 3.19 | -.20 |
| I wanted to keep myself in shape. | 1.43 | .90 | 1.62 | 1.06 | 3.12 | -.19 |
| I wanted to humiliate the person. | 1.06 | .31 | 1.13 | .42 | 2.92 | -.19 |
| It was marital duty. | 1.67 | 1.14 | 1.89 | 1.20 | 2.89 | -.19 |
| The person was a good dancer. | 1.38 | .81 | 1.54 | .91 | 2.95 | -.19 |
| He/she offered me something in exchange for sex. | 1.1 | .40 | 1.2 | .67 | 3.18 | -.18 |
| I was under the influence of porn. | 1.9 | 1.02 | 2.09 | 1.09 | 2.91 | -.18 |
| I wanted to become one with another. | 3.35 | 1.40 | 3.07 | 1.42 | -3.07 | .20 |
| It was a rebellion against parental rules. | 1.25 | .71 | 1.13 | .47 | -2.96 | .20 |
| I wanted to feel loved. | 1.97 | 1.17 | 1.74 | 1.07 | -3.10 | .21 |
| I wanted to belong someone. | 2.23 | 1.33 | 1.97 | 1.18 | -3.13 | .21 |
| I wanted to feel connected to the person. | 2.7 | 1.38 | 2.42 | 1.30 | -3.27 | .21 |
| Due to care. | 2.33 | 1.26 | 2.07 | 1.24 | -3.18 | .21 |
| Due to deepening the relationship. | 3.16 | 1.36 | 2.87 | 1.36 | -3.23 | .21 |
| I was in love. | 4.2 | 1.12 | 3.95 | 1.18 | -3.36 | .22 |
| I wanted to recharge myself. | 2.69 | 1.36 | 2.39 | 1.29 | -3.51 | .23 |
| I didn’t want to ‘‘lose’’ the person. | 1.85 | 1.05 | 1.62 | .95 | -3.50 | .23 |
| I wanted to get closer to the person. | 2.87 | 1.31 | 2.57 | 1.29 | -3.58 | .23 |
| I wanted to balance my mind. | 2.01 | 1.10 | 1.76 | .99 | -3.64 | .24 |
| It was passion. | 3.8 | 1.22 | 3.45 | 1.34 | -4.39 | .27 |
| I wanted to find support. | 1.81 | 1.07 | 1.5 | .92 | -4.59 | .31 |
| I wanted to intensify my relationship. | 2.97 | 1.38 | 2.52 | 1.31 | -5.22 | .33 |
| Due to commitment. | 2.33 | 1.46 | 1.86 | 1.24 | -5.15 | .35 |
| Due to safety seeking. | 2.01 | 1.26 | 1.61 | 1.01 | -5.27 | .35 |
| I was afraid my partner would leave me. | 1.64 | .94 | 1.32 | .66 | -5.85 | .39 |
| Due to trust. | 3.22 | 1.54 | 2.59 | 1.47 | -6.48 | .42 |
| Due to badinage. | 3.38 | 1.36 | 2.74 | 1.40 | -7.25 | .46 |
| Note: Absolute range. 1–5, * p< .005 | | | | | | |
