# Supplementary material for: Why Hungarians Have Sex (YSEX?-HSF)
Source: Arch Sex Behav. 2021 Nov 12;51(1):465–89. doi: 10.1007/s10508-021-02072-y (PMC8858278; doi:10.1007/s10508-021-02072-y)
Supplement: Supplementary file 2 — Supplementary file2 (DOCX 16 kb) [file 10508_2021_2072_MOESM2_ESM.docx]

Supplement 2

*Internal consistency of the 144 item YSEX?-H*

Coefficients of the composites varied between .94 and .96 in each of the male, female and overall samples, which indicates high internal consistency. At the subfactor level in the overall sample, coefficients varied between .67 and .91 within the *Personal Goal Attainment* factor, between .62 and .93 within the *Relational Reasons* factor, and between .62 and .91 within the *Sex as Coping* factor. In the male sample, coefficients of the subfactors varied between .61 and .92, while they varied between .62 and .92 in the female sample.

| Factor | Subfactor | Number of items | Combined  *N* = 1161 | Men  *N* = 341 | Women  *N* = 820 |
| --- | --- | --- | --- | --- | --- |
| Personal Goal Attainment |  | 46 | .95 | .95 | .94 |
|  | Novelty seeking | 12 | .91 | .91 | .90 |
|  | Conformity | 5 | .78 | .80 | .75 |
|  | Infidelity | 5 | .84 | .85 | .82 |
|  | Impulsiveness | 4 | .77 | .76 | .77 |
|  | Revenge | 4 | .76 | .79 | .74 |
|  | Sensation Seeking | 4 | .83 | .84 | .81 |
|  | Control and Power | 6 | .67 | .73 | .63 |
|  | Self-Esteem Boost | 6 | .85 | .86 | .84 |
| Relational Reasons |  | 55 | .96 | .96 | .96 |
|  | Sexual Desire | 11 | .93 | .92 | .92 |
|  | Commitment | 9 | .88 | .87 | .88 |
|  | Physical Attraction | 7 | .85 | .86 | .84 |
|  | Relaxation | 4 | .82 | .79 | .81 |
|  | Intimacy | 5 | .77 | .83 | .76 |
|  | Excitement | 6 | .81 | .84 | .79 |
|  | Self-Affirmation | 6 | .79 | .80 | .79 |
|  | Care | 3 | .72 | .71 | .71 |
|  | Happiness Seeking | 4 | .62 | .66 | .67 |
| Sex as Coping |  | 43 | .94 | .94 | .94 |
|  | Mitigating Emotional Deficit | 12 | .91 | .90 | .90 |
|  | Compulsion and Avoidance | 4 | .73 | .74 | .72 |
|  | Utilitarianism | 7 | .71 | .74 | .69 |
|  | Coping with Relational Conflicts | 6 | .81 | .74 | .78 |
|  | Submissiveness | 4 | .62 | .61 | .62 |
|  | Dealing with Partner’s Emotional Demands | 5 | .72 | .82 | .64 |
|  | Mate Retention | 5 | .86 | .81 | .88 |
| *Note*: Internal consistency scores are Cronbach’s coefficient alphas. | | | | | |
