# Supplementary material for: Why Hungarians Have Sex (YSEX?-HSF)
Source: Arch Sex Behav. 2021 Nov 12;51(1):465–89. doi: 10.1007/s10508-021-02072-y (PMC8858278; doi:10.1007/s10508-021-02072-y)
Supplement: Supplementary file 3 — Supplementary file3 (DOCX 18 kb) [file 10508_2021_2072_MOESM3_ESM.docx]

Supplement 3

*Spearman’s rank correlations (ρ) between age and the factors and subfactors of the YSEX?-H questionnaire*

Correlations between age and the factors and subfactors of YSEX-H were tested by calculating Spearman’s rank correlation coefficients for each gender group and for the overall sample (significance was tested at *p* < .05 and *p* < .01; see Table 10). The results revealed a characteristic pattern of correlations between age and the factors and subfactors. Various sexual motives generally showed (weak) negative correlations with age, except the Infidelity subfactor, which showed a positive correlation. The Personal Goal Attainment factor did not correlate significantly with age in the overall sample (while these two variables showed a significant negative correlation in each gender group) probably because the negative correlations obtained for Impulsiveness and Self-Esteem Boost counterbalanced the positive correlation obtained for Infidelity. All subfactors of the Relational Reasons factor showed a significant negative correlation with age in the overall sample. Although the Sex as Coping factor showed a significant negative correlation with age in the overall sample, no significant correlation was found between these two variables in either gender group. Moreover, significant (weak) negative correlations for some of the subfactors were found in the female subsample (Emotional Need Satisfaction, Coping with Relational Conflicts), and (Utilitarianism, Submission) in the male subsample.

| Factor and Subfactor (144 items) | *ρ* | | | | | |
| --- | --- | --- | --- | --- | --- | --- |
|  | Overall  (*n* = 1161) | | Women  (*n* = 820) | | Men  (*n* = 341) | |
| Personal Goal Attainment | -.05 |  | -.11^*^ |  | -.15^**^ |  |
| Novelty Seeking | -.037 |  | -.11^*^ | ^­^ | -.14^**^ |  |
| Conformity | .00 |  | -.04 |  | -.12^*^ |  |
| Infidelity | .25^**^ |  | .20^**^ |  | .18^**^ |  |
| Impulsiveness | -.11^**^ |  | -.13^**^ |  | -.20^**^ |  |
| Revenge | .01 |  | .04 |  | .01 |  |
| Sensation Seeking | -.06^*^ |  | -.14^**^ |  | -.12^*^ |  |
| Control and Power | -.06^*^ |  | -.07^*^ |  | -.18^**^ |  |
| Self-Esteem Boost | -.11^*^ |  | -.11^**^ |  | -18^**^ |  |
| Relational Reasons | -.18^**^ |  | -.16^**^ |  | -.22^**^ |  |
| Sexual Desire | -.12^**^ |  | -.13^**^ |  | -.22^**^ |  |
| Commitment | -.19^**^ |  | -.13^*^ |  | -.17^**^ |  |
| Physical Attraction | -.12^**^ |  | -.18^**^ |  | -.16^**^ |  |
| Relaxation | -.18^**^ |  | -.15^**^ |  | -.21^**^ |  |
| Intimacy | -.14^**^ |  | -.10^*^ |  | -.13^*^ |  |
| Excitement | -.14^**^ |  | -.16^**^ |  | -.17^**^ |  |
| Self-Affirmation | -.09^**^ |  | -.04 |  | -.11^*^ |  |
| Care | -.25^**^ |  | -.24^**^ |  | -.25^**^ |  |
| Happiness Seeking | -.13^**^ |  | -.12^*^ |  | -.12^*^ |  |
| Sex as Coping | -.09^**^ |  | -.05 |  | -.09 |  |
| Mitigating Emotional Deficit | -.11^**^ |  | -.07^*^ |  | -.09 |  |
| Compulsion and Avoidance | .01 |  | .03 |  | -.05 |  |
| Utilitarianism | -.08^**^ |  | .03 |  | -.15^**^ |  |
| Coping with Relational Conflicts | -.11^*^ |  | -.09^**^ |  | -.11 |  |
| Submission | -.10^**^ |  | -.05 |  | -.12^*^ |  |
| Dealing with Partner’s Emotional Needs | .02 |  | -.04 |  | .02 |  |
| Mate Retention | -.03 |  | .01 |  | .04 |  |
| *Note*: * *p* < .05, ** *p* < .001 | | | | | | |
