# Supplementary material for: Why Hungarians Have Sex (YSEX?-HSF)
Source: Arch Sex Behav. 2021 Nov 12;51(1):465–89. doi: 10.1007/s10508-021-02072-y (PMC8858278; doi:10.1007/s10508-021-02072-y)
Supplement: Supplementary file 4 — Supplementary file4 (DOCX 14 kb) [file 10508_2021_2072_MOESM4_ESM.docx]

Supplement 4

*Descriptive data and the adapted version of the Kinsey Scale*

The distribution of relationship statuses in the sample was as follows: currently single: 2.7% (women: 3.5%, men: 1.8%), casual sexual contacts without a permanent relationship: 13.0% (women: 13.3%, men: 12.6%), dating someone (but not living together): 7.3% (women: 8.1%, men: 6.3%), domestic partnership: 20.1% (women: 22.7%, men: 16.8%), married: 30.2% (women: 34.1%, men: 25.1%). The ‘other, specifically’ response category was selected by 26.7% (women: 18.3%, men: 37.4%). These respondents described specific statuses such as ‘I am currently dating a woman with two kids, she is 37 and has still not got divorced, I think our relationship will never go deeper’; ‘I am still married, unable to get divorced, living with someone else for one and a half years’; ‘I lived together with someone from whom I have moved away, but whom I am still intimately attached to, although I have sex with others and not with him’; ‘having a friend with benefits’; ‘going to be married in two months’; ‘I am a lover’; ‘an intense emotional and sexual affair going on for long but as an open relationship – we do not live together, and we both date others at night’.

Respondents had been in their current relationship for 70.07 months on average (*SD* = 94.35, range: 1-612). Women had been in their current relationship for 51.46 months (*SD* = 78.03, range: 1-612) and men for 94.19 months (*SD* = 107.41, range: 1-540). The distribution of relationship statuses among those currently having a relationship was as follows: dating someone (for less than 6 months): 7.9% (women: 9.0%, men: 6.5%), permanent relationship / married: 72.2% (women: 71.1%, men: 74.9%).

Most researchers studying human sexual behavior use Kinsey’s scale to assess sexual orientation (e.g. Kinsey, Pomeroy & Martin, 1948; Kinsey, Pomeroy, Martin & Gebhard, 1953). In our view, however, this scale only provides limited information on its subject, since it focuses on only one aspect of a complex phenomenon, namely, on respondents’ identity related to their sexual orientation. Since we were also interested in respondents’ behavioral experience and sexual desire, they received three questions related to their sexual orientation, which they answered using a 7-point scale in each case as follows.

(A) Please indicate the extent to which you are interested in a sexual partner of the same and/or the opposite gender.

1. I am exclusively interested in a sexual partner of the opposite gender.

2. I am predominantly interested in a sexual partner of the opposite gender, but sometimes I am also interested in one of the same gender.

3. I prefer a sexual partner of the opposite gender, but I am often interested in one of the same gender.

4. I am equally interested in a sexual partner of the same and the opposite gender.

5. I prefer a sexual partner of the same gender, but I am often interested in one of the opposite gender.

6. I am predominantly interested in a sexual partner of the same gender, but sometimes I am also interested in one of the opposite gender.

7. I am exclusively interested in a sexual partner of the same gender.

(B) Please indicate how frequently you have had sexual experience with a partner of the same and/or the opposite gender.

1. I have exclusively had sexual experience with a partner of the opposite gender.

2. I have predominantly had sexual experience with a partner of the opposite gender, but I have also had some experience with a partner of the same gender.

3. I have had more sexual experience with a partner of the opposite gender, but I have also had frequent experience with a partner of the same gender.

4. I have equally had sexual experience with a partner of the same and the opposite gender.

5. I have had more sexual experience with a partner of the same gender, but I have also had frequent experience with a partner of the opposite gender.

6. I have predominantly had sexual experience with a partner of the same gender, but I have also had some experience with a partner of the opposite gender.

7. I have exclusively had sexual experience with a partner of the same gender.

(C) Please indicate which of the below definitions best describes your sexual orientation.

0. Exclusively heterosexual

1. Predominantly heterosexual, only incidentally homosexual

2. Predominantly heterosexual, but more than incidentally homosexual

3. Equally heterosexual and homosexual

4. Predominantly homosexual, but more than incidentally heterosexual

5. Predominantly homosexual, only incidentally heterosexual

6. Exclusively homosexual

The following findings were obtained with these three questions. Of the overall sample, 78.0% were exclusively interested in a sexual partner of the opposite gender (women: 68.7%, men: 90.1%); 18.8% were predominantly interested in a sexual partner of the opposite gender but sometimes also interested in one of the same gender (women: 27.5%, men: 7.4%); 1.7% preferred a sexual partner of the opposite gender, but they were often interested in one of the same gender (women: 2.2%, men: 0.9%); all other response alternatives were selected by 1.6% in total (women: 1.5%, men: 1.6%). Of the overall sample, 82.2% had exclusively had sexual experience with a partner of the opposite gender (women: 75.6%, men: 90.8%); 15.0% had predominantly had sexual experience with a partner of the opposite gender, but they had also had some experience with a partner of the same gender (women: 21.3%, men: 7.0%); 1.2% had had more sexual experience with a partner of the opposite gender, but they had also had frequent experience with a partner of the same gender (women: 1.4%, men: 0.9%); all other response alternatives were selected by 1.6% in total (women: 1.7%, men: 1.2%). Of the overall sample, 81.3% reported to be exclusively heterosexual (women: 74.7%, men: 89.9%); 15.9% to be predominantly heterosexual, only incidentally homosexual (women: 21.8%, men: 8.3%); 1.3% to be predominantly heterosexual but more than incidentally homosexual (women: 1.7%, men: 0.7%); all other response alternatives were selected by 1.5% in total (women: 1.7%, men: 1.1%). The relationship between sexual orientation and sexual motivation falls outside the scope of the present paper. A separate paper will be dedicated to this subject.
