# Supplementary material for: Why Hungarians Have Sex (YSEX?-HSF)
Source: Arch Sex Behav. 2021 Nov 12;51(1):465–89. doi: 10.1007/s10508-021-02072-y (PMC8858278; doi:10.1007/s10508-021-02072-y)
Supplement: Supplementary file 5 — Supplementary file5 (DOCX 25 kb) [file 10508_2021_2072_MOESM5_ESM.docx]

Supplement 5

*Hungarian Version and Short Form of Reasons for Having Sex Questionnaire (YSEX?-HSF)*

People have sex (i.e., sexual intercourse) for many different reasons. Below is a list of some of these reasons. Please indicate how frequently each of the following reasons led you to have sex in the past. For example, if about half of the time you engaged in sexual intercourse you did so because you were bored, then you would circle “3” beside question 4. If you have not had sex in the past, use the following scale to indicate what the likelihood that each of the following reasons would lead you to have sex.

I have had sex in the past because...

| 1 | 2 | 3 | 4 | 5 |
| --- | --- | --- | --- | --- |
| None of my sexual experiences | A few of my sexual experiences | Some of my sexual experiences | Many of my sexual experiences | All of my sexual experiences |

| Item # | Item Description | Rating | | | | |
| --- | --- | --- | --- | --- | --- | --- |
| 1. I wanted a new experience. | | 1 | 2 | 3 | 4 | 5 |
| 2. I love variety. | | 1 | 2 | 3 | 4 | 5 |
| 3. It was forbidden. | | 1 | 2 | 3 | 4 | 5 |
| 4. It was trendy. | | 1 | 2 | 3 | 4 | 5 |
| 5. I didn’t want to be the odd one out. | | 1 | 2 | 3 | 4 | 5 |
| 6. I wanted to be cool. | | 1 | 2 | 3 | 4 | 5 |
| 7. My partner did not want to have sex with me (so I had sex with someone else). | | 1 | 2 | 3 | 4 | 5 |
| 8. My partner was not able to have sex with me (so I had sex with someone else). | | 1 | 2 | 3 | 4 | 5 |
| 9. My partner is not adventurous enough (so I had sex with someone else). | | 1 | 2 | 3 | 4 | 5 |
| 10. I was under the influence of alcohol. | | 1 | 2 | 3 | 4 | 5 |
| 11. I was in an altered state of consciousness. | | 1 | 2 | 3 | 4 | 5 |
| 12. We hooked up in the heat of the moment. | | 1 | 2 | 3 | 4 | 5 |
| 13. I wanted I wanted to infuriate someone. | | 1 | 2 | 3 | 4 | 5 |
| 14. I wanted to take revenge. | | 1 | 2 | 3 | 4 | 5 |
| 15. I wanted to make my partner jealous. | | 1 | 2 | 3 | 4 | 5 |
| 16. I wanted to seek experience. | | 1 | 2 | 3 | 4 | 5 |
| 17. It was a seduction/I was seduced. | | 1 | 2 | 3 | 4 | 5 |
| 18. I took the opportunity. | | 1 | 2 | 3 | 4 | 5 |
| 19. I wanted to control the other person. | | 1 | 2 | 3 | 4 | 5 |
| 20. I wanted to demonstrate my power. | | 1 | 2 | 3 | 4 | 5 |
| 21. Out of a desire for possession. | | 1 | 2 | 3 | 4 | 5 |
| 22. To prove myself. | | 1 | 2 | 3 | 4 | 5 |
| 23. I wanted to boost my self-esteem. | | 1 | 2 | 3 | 4 | 5 |
| 24. I wanted to feel appreciated. | | 1 | 2 | 3 | 4 | 5 |
| 25. I wanted to have an orgasm. | | 1 | 2 | 3 | 4 | 5 |
| 26. Because of sexual desire. | | 1 | 2 | 3 | 4 | 5 |
| 27. I wanted pleasure. | | 1 | 2 | 3 | 4 | 5 |
| 28. It seemed like the next step in the relationship. | | 1 | 2 | 3 | 4 | 5 |
| 29. I wanted to deepen the relationship. | | 1 | 2 | 3 | 4 | 5 |
| 30. I wanted the relationship to grow. | | 1 | 2 | 3 | 4 | 5 |
| 31. The person had an attractive body. | | 1 | 2 | 3 | 4 | 5 |
| 32. The person smelled good. | | 1 | 2 | 3 | 4 | 5 |
| 33. The person had an attractive personality. | | 1 | 2 | 3 | 4 | 5 |
| 34. I wanted to feel fresh. | | 1 | 2 | 3 | 4 | 5 |
| 35. I wanted to reduce stress. | | 1 | 2 | 3 | 4 | 5 |
| 36. I wanted to recharge myself. | | 1 | 2 | 3 | 4 | 5 |
| 37. I was in love. | | 1 | 2 | 3 | 4 | 5 |
| 38. We were made for each other. | | 1 | 2 | 3 | 4 | 5 |
| 39. Out of passion. | | 1 | 2 | 3 | 4 | 5 |
| 40. I wanted to have sex in an unusual place or situation. | | 1 | 2 | 3 | 4 | 5 |
| 41. It was a special occasion/situation. | | 1 | 2 | 3 | 4 | 5 |
| 42. I wanted to try out new sexual techniques or positions. | | 1 | 2 | 3 | 4 | 5 |
| 43. I wanted to spiritually merge with the other person. | | 1 | 2 | 3 | 4 | 5 |
| 44. I wanted to express myself. | | 1 | 2 | 3 | 4 | 5 |
| 45. To get on the same wavelength with the other person. | | 1 | 2 | 3 | 4 | 5 |
| 46. I wanted to care for the other person. | | 1 | 2 | 3 | 4 | 5 |
| 47. I wanted to cheer up the other person. | | 1 | 2 | 3 | 4 | 5 |
| 48. I wanted to celebrate. | | 1 | 2 | 3 | 4 | 5 |
| 49. I wanted to make my partner happy. | | 1 | 2 | 3 | 4 | 5 |
| 50. I wanted to be/feel happy. | | 1 | 2 | 3 | 4 | 5 |
| 51. The touch of the person was pleasant. | | 1 | 2 | 3 | 4 | 5 |
| 52. I wanted to decrease loneliness. | | 1 | 2 | 3 | 4 | 5 |
| 53. Because I lacked love. | | 1 | 2 | 3 | 4 | 5 |
| 54. I wanted to decrease sadness. | | 1 | 2 | 3 | 4 | 5 |
| 55. Out of duty. | | 1 | 2 | 3 | 4 | 5 |
| 56. Out of habit/routine. | | 1 | 2 | 3 | 4 | 5 |
| 57. I wanted to avert my partner’s suspicion. | | 1 | 2 | 3 | 4 | 5 |
| 58. I wanted to profit from it. | | 1 | 2 | 3 | 4 | 5 |
| 59. It was a way to reach my goal. | | 1 | 2 | 3 | 4 | 5 |
| 60. I wanted to benefit from it. | | 1 | 2 | 3 | 4 | 5 |
| 61. I wanted to apologize. | | 1 | 2 | 3 | 4 | 5 |
| 62. I wanted to make peace with the other person. | | 1 | 2 | 3 | 4 | 5 |
| 63. I wanted to appease the other person. | | 1 | 2 | 3 | 4 | 5 |
| 64. I wanted to submit myself. | | 1 | 2 | 3 | 4 | 5 |
| 65. I wanted to seek safety. | | 1 | 2 | 3 | 4 | 5 |
| 66. I wanted to prove sg to my partner. | | 1 | 2 | 3 | 4 | 5 |
| 67. I wanted to give in. | | 1 | 2 | 3 | 4 | 5 |
| 68. It was a favor. | | 1 | 2 | 3 | 4 | 5 |
| 69. Out of compassion. | | 1 | 2 | 3 | 4 | 5 |
| 70. I wanted to comfort the other person. | | 1 | 2 | 3 | 4 | 5 |
| 71. I wanted to retain the relationship. | | 1 | 2 | 3 | 4 | 5 |
| 72. I wanted to save the relationship. | | 1 | 2 | 3 | 4 | 5 |
| 73. Not to lose the other person. | | 1 | 2 | 3 | 4 | 5 |

Scoring System

| Scale | Subscale | Item Numbers | Score Range |
| --- | --- | --- | --- |
| Personal goal attainment |  | 1-24 | 8-40 |
|  | Novelty seeking | 1-3 | 3-15 |
|  | Conformity | 4-6 | 3-15 |
|  | Infidelity | 7-9 | 3-15 |
|  | Impulsiveness | 10-12 | 3-15 |
|  | Revenge | 13-15 | 3-15 |
|  | Sensation seeking | 16-18 | 3-15 |
|  | Control and power | 19-21 | 3-15 |
|  | Self-esteem boost | 22-24 | 3-15 |
| Relational reasons |  | 25-51 | 9-45 |
|  | Sexual desire | 25-27 | 3-15 |
|  | Commitment | 28-30 | 3-15 |
|  | Physical attraction | 31-33 | 3-15 |
|  | Relaxation | 34-36 | 3-15 |
|  | Intimacy | 37-39 | 3-15 |
|  | Excitement | 40-42 | 3-15 |
|  | Self-affirmation | 43-45 | 3-15 |
|  | Care | 46-48 | 3-15 |
|  | Happiness seeking | 49-51 | 3-15 |
| Sex as coping |  | 52-73 | 7-35 |
|  | Mitigating emotional deficit | 52-54 | 3-15 |
|  | Compulsion and avoidance | 55-57 | 3-15 |
|  | Utilitarianism | 58-60 | 3-15 |
|  | Coping with relational conflicts | 61-63 | 3-15 |
|  | Submissiveness | 64-67 | 4-20 |
|  | Coping with partner’s emotional demands | 68-70 | 3-15 |
|  | Mate retention | 71-73 | 3-15 |

*Note*: Subscale scores are computed by adding the scores of the individual items that comprise the subscale. Scale scores are computed by adding the scores of the items that comprise each of the subscale under that specific factor.
